# Interrogation of gender disparity uncovers androgen receptor as the transcriptional activator for oncogenic *miR-125b* in gastric cancer

**DOI:** 10.1038/s41419-021-03727-3

**Published:** 2021-05-04

**Authors:** Ben Liu, Meng Zhou, Xiangchun Li, Xining Zhang, Qinghua Wang, Luyang Liu, Meng Yang, Da Yang, Yan Guo, Qiang Zhang, Hong Zheng, Qiong Wang, Lian Li, Xinlei Chu, Wei Wang, Haixin Li, Fengju Song, Yuan Pan, Wei Zhang, Kexin Chen

**Affiliations:** 1 Department of Epidemiology and Biostatistics, Tianjin Medical University Cancer Institute and Hospital, Tianjin, People’s Republic of China; 2 Tianjin Cancer Institute, Tianjin Medical University Cancer Institute and Hospital, Tianjin, People’s Republic of China; 3 Cancer Institute, The Second Hospital of Dalian Medical University, Dalian, People’s Republic of China; 4 Center for Pharmacogenetics, Department of Pharmaceutical Sciences, Department of Computational and Systems Biology University of Pittsburgh Cancer Institute, University of Pittsburgh, Pittsburgh, PA 15261 USA; 5 Department of Cancer Biobank, Tianjin Medical University Cancer Institute and Hospital, Tianjin, People’s Republic of China; 6 Department of Maxillofacial and Otorhinolaryngology Oncology, Tianjin Medical University Cancer Institute and Hospital, Tianjin, People’s Republic of China; 7 Department of Senior Ward, National Clinical Research Center for Cancer, Key Laboratory of Molecular Cancer Epidemiology of Tianjin, Tianjin Medical University Cancer Institute and Hospital, Tianjin, People’s Republic of China; 8 Wake Forest Baptist Comprehensive Cancer Center, Wake Forest Baptist Medical Center, Winston- Salem, NC USA; 9 Department of Cancer Biology, Wake Forest School of Medicine, Winston-Salem, NC USA

**Keywords:** Tumour biomarkers, Translational research

## Abstract

There is a male preponderance in gastric cancer (GC), which suggests a role of androgen and androgen receptor (AR). However, the mechanism of AR signaling in GC especially in female patients remains obscure. We sought to identify the AR signaling pathway that might be related to prognosis and examine the potential clinical utility of the AR antagonist for treatment. Deep learning and gene set enrichment analysis was used to identify potential critical factors associated with gender bias in GC (*n* = 1390). Gene expression profile analysis was performed to screen differentially expressed genes associated with AR expression in the Tianjin discovery set (*n* = 90) and TCGA validation set (*n* = 341). Predictors of survival were identified via lasso regression analyses and validated in the expanded Tianjin cohort (*n* = 373). In vitro and in vivo experiments were established to determine the drug effect. The GC gender bias was attributable to sex chromosome abnormalities and AR signaling dysregulation. The candidates for AR-related gene sets were screened, and AR combined with miR-125b was associated with poor prognosis, particularly among female patients. AR was confirmed to directly regulate *miR-125b* expression. AR-miR-125b signaling pathway inhibited apoptosis and promoted proliferation. AR antagonist, bicalutamide, exerted anti-tumor activities and induced apoptosis both in vitro and in vivo, using GC cell lines and female patient-derived xenograft (PDX) model. We have shed light on gender differences by revealing a hormone-regulated oncogenic signaling pathway in GC. Our preclinical studies suggest that AR is a potential therapeutic target for this deadly cancer type, especially in female patients.

## Introduction

Gastric cancer (GC) is the fifth most frequently diagnosed cancer and the third leading cause of cancer-related deaths in both sexes globally and more common in East Asia^1^. In all populations studied worldwide, there is a male preponderance in GC with an age-standardized incidence ratio of 2:1 for male versus female. There is a higher survival rate in females after treatment^1,2^. Although better insights have been devoted to study genetic variation and molecular signatures of GC^3–6^, we still have a long-standing problem in the understanding of the molecular mechanism and phenotypic variation among individuals^7^, especially between genders^8,9^.

An enigmatic male predominance characterizes the incidence of GC. However, this sex disparity cannot be entirely attributed to the differences in the prevalence of known risk factors between the genders^10–14^. However, only a few studies focused on female patients with GC^15^, and female sex as a prognostic factor for GC remains controversial^16^. Concentrations of sex steroid hormones and sex hormone receptors have been hypothesized to explain this sex disparity^17^.

Androgen receptor (AR), as a member of the intracellular nuclear receptor subfamily, is involved in many physiological functions as a ligand-dependent transcriptional factor^18–20^. Despite some indications linking AR to the occurrence and progression of gastric tumors^21–23^, considerable controversy exists concerning AR expression levels and prognostic value in GC^14,23,24^. Another dilemma is whether the AR expression pattern and AR-regulated signaling pathway is correlated with the sex disparity of GC^25,26^.

In this study, we found AR played an important role in gender difference by proposing a new deep learning model and demonstrated that AR is an upstream transcriptional regulator of miR-125b^27–29^, which exhibits an oncogenic potential in GC. Hormone therapy by targeting AR may be a treatment option for GC.

## Results

### Androgen receptor (AR) was predicted as key contributing factors in gender-specific difference

**Fig. 1:**
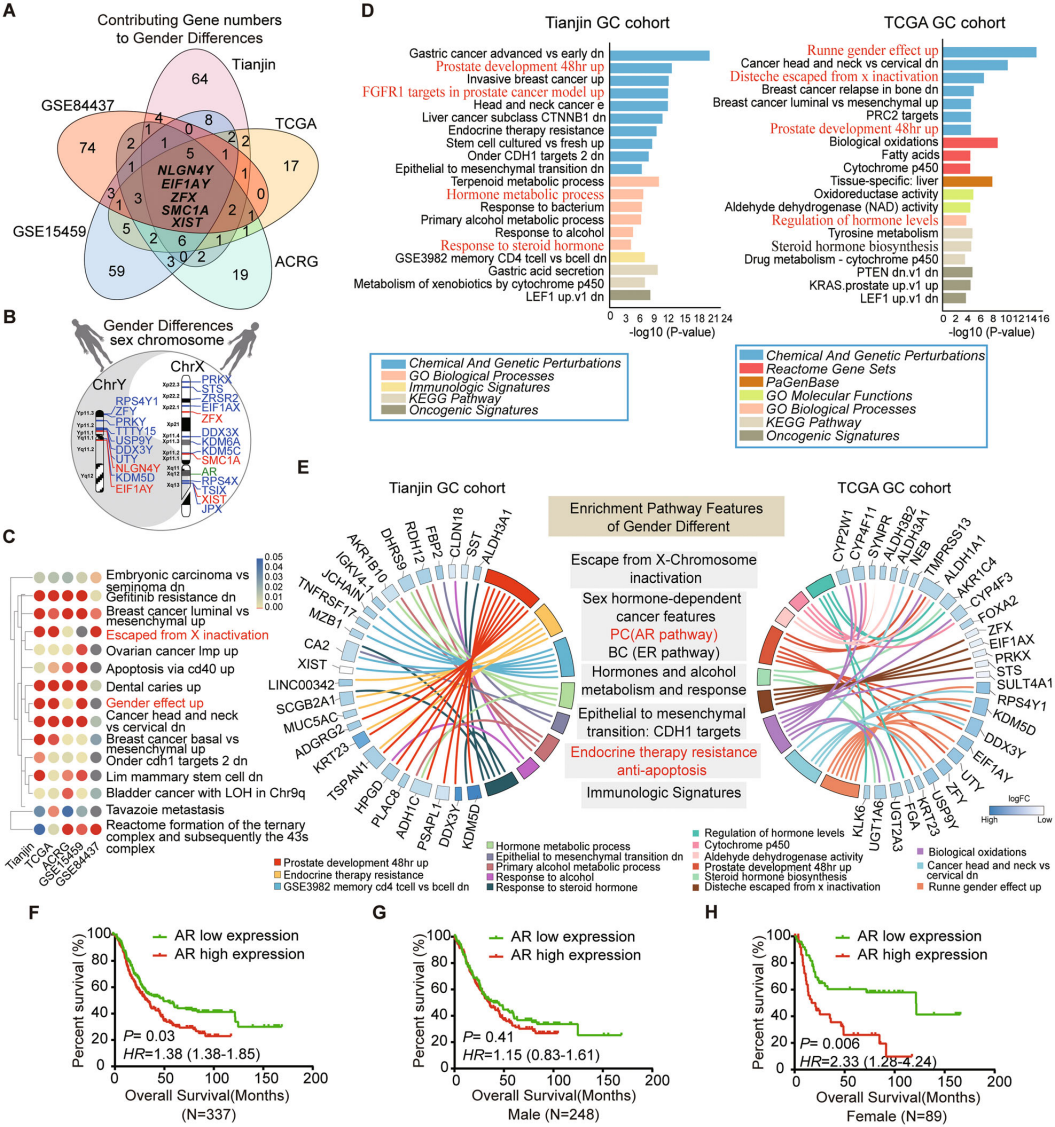
Prediction of candidate factors to cause gender bias in GC. **A** The Venn diagram was showing overlapping gene numbers of the top 100 high contribution value (attribution score) to gender difference identified from the five GC cohorts (Tianjin, TCGA, ACRG, GSE15459, and GSE84437) by using an algorithm of deep learning. The intersection of the Venn diagram shows the number and the name of overlapping genes among all five datasets. **B** The schematic diagram for overlapping gender bias-related gene locus in all five gene sets on sex chromosome X and Y by deep learning. Red represents the intersection of all five gene sets; blue represents the intersection of 4 or 3 GC gene sets; green represents the Androgen receptor (AR) on chromosome X. **C** The heatmap visualization of top 15 enriched terms across five gender- bias associated gene sets of GC using deep learning method. *P* < 0.05. Red terms represent candidate factors proposed to cause gender bias include X-chromosome abnormalities and sex hormones. **D** Bar plot ranking of the top 20 clusters by Metascape pathway enrichment analysis of differentially expressed genes between genders in Tianjin (left panel) and TCGA (right panel) datasets. Red terms highlight the X Chromosomal abnormalities, sex hormone, and hormone receptor-associated pathway and process. **E** Circos plot demonstrating the relationship between selected Metascape clusters and their genes in Tianjin (left) and TCGA (right) dataset. Genes are located on the left (Tianjin circos) or right (TCGA circos) side of the graph and indicated by their symbols. The logFC values of genes are exhibited by heatmap. Gene involvement in the Metascape clusters was determined by connecting lines. The representative pathways and biological process features, which were related to gender differences, are listed in gray background boxes in the middle panel. PC: prostate cancer, BC: breast cancer. **F** Kaplan–Meier curves for overall survival of 337 GC patients in Tianjin cohort. **G** and **H** Kaplan–Meier analysis of overall survival according to low and high AR expression in 248 male (**G**) and in 89 female (**H**) cases.

To find the candidate factors to cause gender bias in GC, we used two different strategies. First, we developed a machine-learning-based algorithm that permits a genome-wide total of 20,706 genes to enter the model using five large GC datasets (including Tianjin, TCGA, ACRG, GSE15459, and GSE84437) (Fig. 1A-C). We evaluated and ranked the relative importance of each gene expression (attribution score) in gender difference. The 100 highest ranked genes in each dataset were analyzed for shared genes (Fig. 1A and Supplementary Table S2). The result showed including five genes shared in all five datasets, the vast majority of overlapping genes were localized on sex chromosomes (Fig. 1B). The AR gene was also mapped on the X-chromosome as an important sex hormone receptor linked to the above genes in Fig. 1B. Furthermore, the GSEA analysis using the same deep learning algorithm showed these predicted genes were associated with sex chromosome abnormalities and some sex hormone signaling pathways, such as escaping from X-chromosome inactivation and gender effect up (Fig. 1C).

Second, we perform function and pathway enrichment analyses based on the online bioinformatics database Metascape (https://metascape.org/gp/index.html#/main/step1) in Tianjin and TCGA GC dataset (Fig. 1D, E). Unlike deep learning methods, only differentially expressed genes (DEGs) between genders were included in this analysis. Several driving factors and the specific biological pathways for gender differences were exhibited, such as escape from X-chromosome, sex hormone- dependant cancer features (prostate cancer and breast cancer), steroid hormone, alcohol metabolism/response well as EMT and immune-related pathways (Fig. 1E). All of these results together suggested AR is a key candidate gene in the gender bias of GC.

### AR combined with miR-125b is associated with poor prognosis in GC

To gain insight into the clinical relevance of AR expression in GC, we performed survival analysis in the expanded Tianjin GC cohort analysis of 337 patients. The results showed that patients with high AR expression had poorer overall survival (OS) and disease-free survival (DFS) compared to those with low expression (Log-rank test *P* = 0.03 and 0.02, respectively) (Fig. 1F and Supplementary Fig. S1). Furthermore, these significant differences in prognosis were only found in female (*P* = 0.006 of OS and *P* = 0.002 of DFS) but not in male cases (*P* = 0.41 of OS and *P* = 0.42 of DFS) (Fig. 1G, H and Supplementary Fig. S1). Cox regression analysis showed that AR expression was significantly associated with shorter OS (HR, 1.38; 95% CI, 1.02–1.85) and DFS (HR, 1.46; 95% CI, 1.10–1.94) after adjusting for confounding variables (Supplementary Table S3). Higher expression levels of AR were also associated with tumor size and lymph node (LN) metastasis (*P* = 0.000 and 0.006, respectively) (Supplementary Table S2). Higher expression levels of AR were also associated with tumor size (Chi-square test, *P* = 0.000) and LN metastasis (Chi-square test, *P* = 0.006) (Supplementary Table S1).

**Fig. 2:**
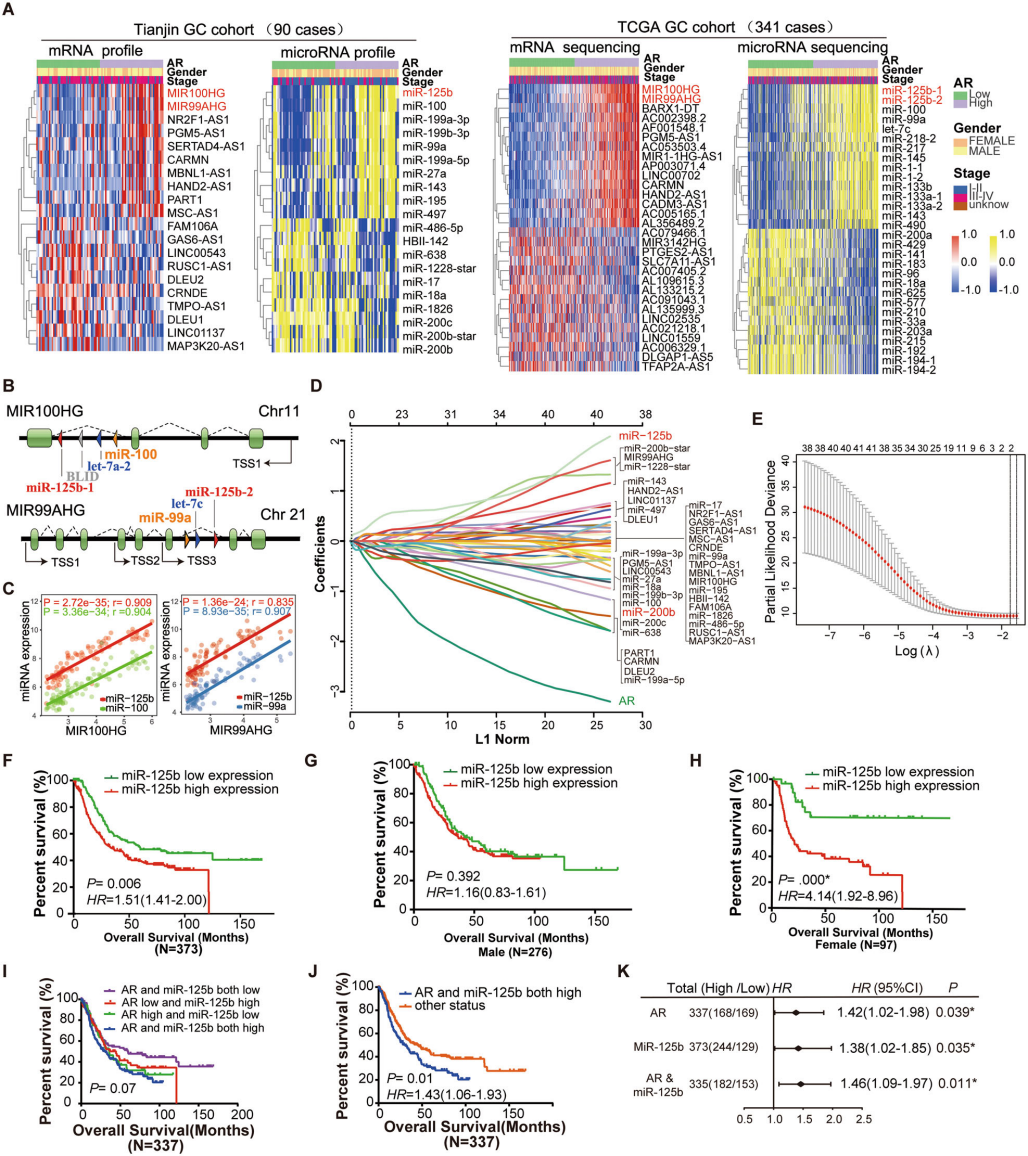
Identification of key genes associated with AR expression and the prognostic value validation of the AR-related gene set. **A** The heatmap shows top significantly up-regulated or down-regulated genes (mRNA and micro-RNA) between AR high and low expression across Tianjin (the two panels on the left) and TCGA (the two panels on the right) GC tumor samples. The red and yellow mean up-regulated mRNA and miRNA, respectively. The blue means down-regulated mRNA and miRNAs. Gene name in red indicates key significant lncRNA and miRNA in both GC datasets. **B** Diagrams of lncRNA MIR100HG and MIR99AHG gene structure. MIR100HG: the host gene of miR-100/let-7a-2/BLLD/miR-125b-1 cluster on human chromosome 11 (chr11). MIR99AHG: the host gene of miR-99a/let-7c/miR-125b-2 cluster on human chromosome 21 (chr21). **C** Scatter plots depict the correlation for MIR100HG versus miR-125b or miR-100 (left panel) and MIR99AHG versus miR-125b or miR-99a (right panel) in Tianjin dataset. Scatter plots of expression in TCGA CRC data repository. *R*: Pearson correlation coefficients. **D** and **E** Identification of AR-related gene risk signature for OS by LASSO regression analysis in Tianjin datasets. **D** LASSO coefficient profiles of the 40 AR expression-associated genes (including lncRNA and miRNAs). The gene name in red indicates the significant gene by the LASSO screening process. The numbers of selected covariates were two for the tuning parameter λ. **E** Partial likelihood deviance as a function of parameter estimated over a grid of values for λ. A vertical line is drawn at the value chosen by tenfold. **F–H** Association of expression of miR-125b with gastric cancer survival of 373 cases in Tianjin GC cohorts. **F** Kaplan–Meier analysis of overall survival according to low and high miR-125b expression in 373 cases. **G–H** Kaplan–Meier analysis of overall survival according to low and high miR-125b expression in 276 male (**G**) and 97 female (**H**) cases. Error bars represent ± SD. **I–K** Association of expression of AR and miR-125b with gastric cancer survival. **I** Kaplan–Meier analysis of overall survival in four groups according to low and high AR and miR-125b expression. **J** Kaplan–Meier analysis of overall survival in two groups according to low and high AR and miR-125b expression. In addition to the group of AR/miR-125b both high expressions, the other three groups in the panel (**I**) were merged into one group. **K** Forest plot for the overall survival analysis of AR and/or miR-125b using a multivariable Cox proportional hazards model after adjustment for multiple clinical variables.

By analyzing whole-genome transcriptional microarray profiling data (mRNA and miRNA) of the Tianjin cohort, we further identified the DEGs between AR high and low expression (Fig. 2A, the left two panels). The main results were validated using the independent TCGA dataset (Fig. 2A, the right two panels). It was noteworthy that the common features of gene expression patterns both in Tianjin and TCGA datasets (Fig. 2A-C). The levels of lncRNA *MIR100HG* and *MIR99AHG* showed significantly up-regulated in AR high expression group, which was accompanied by significant up-regulation of *miR-125b*, *miR-100*, and *miR-99a*. Several members of the miR-200 family (*miR-200a*, *b*, *c*, and *miR-141*) and *miR-18a* expression were significantly decreased in AR low expression group (Fig. 2A). The *MIR100HG* and *MIR99AHG* are the host genes of the *miR-100*/*let-7a-2*/*miR-125b*-1 cluster and the *miR-99a*/*let-7c*/*miR-125b*-2 cluster respectively (Fig. 2B). Co-expression analysis confirmed a positive correlation of *MIR100HG* and *MIR99AGH* with *miR-125b* in Tianjin (Fig. 2C) and TCGA dataset (Supplementary Fig. S2).

To identify AR-related gene signature associated with GC outcome, the top 40 differential expressed genes of Tianjin dataset in Fig. 2A were undergone the LASSO regression analysis (Fig. 2D, E). A total of two candidate miRNAs (*miR-125b* and *miR-200b*) (Fig. 2D) were selected as survival-associated featured genes (Fig. 2E). Kaplan–Meier (K–M)survival analysis in the expanded Tianjin GC cohort showed that patients with high *miR-125b* expression had adverse OS and DFS compared to those with low expression (Log-rank test *P* = 0.006 and 0.009, respectively) (Fig. 2F and Supplementary Fig. S3A). There was a significant association between prognosis and *miR-125b* expression in female (Log-rank test *P* = 0.000 in OS and 0.001 in DFS, respectively) (Fig. 2H and Supplementary Fig. S3C), but not in male GC patients (Log-rank test *P* = 0.392 in OS and 0.429 in DFS, respectively) (Fig. 2G and Supplementary Fig. S3B). Cox regression analysis showed that *miR-125b* expression was associated with OS (HR, 1.42; 95% CI, 1.02–1.98) and DFS (HR, 1.27; 95% CI, 0.98–1.66) after adjusting for confounding variables (Supplementary Table S3).

To further evaluate the prognostic value of AR/*miR-125b* in GC, we divided the cases into four groups (AR and *miR-125b* both low; both high; AR high and *miR-125b* low; AR low and *miR-125b* high). We observed a trend that the high expression of AR/*miR-125b* was associated with the poor prognosis of GC (log-rank test *P* = 0.07 in OS and 0.023 in DFS) (Fig. 2I and Supplementary Fig. S3D). The AR and *miR-125b* both high group were associated with poor survival of GC cases comparing with the remaining three groups together (log-rank test *P* = 0.01 in OS and 0.01 in DFS) (Fig. 2J and Supplementary Fig. S3E). The Cox regression analysis showed that AR combining *miR-125b* expression was associated with OS (HR, 1.46; 95% CI, 1.09–1.97) and DFS (HR, 1.50; 95% CI, 1.13–2.00) after adjusting for confounding variables (Fig. 2K and Supplementary Table S3).

### AR transcriptionally activates *miR-125b* expression

**Fig. 3:**
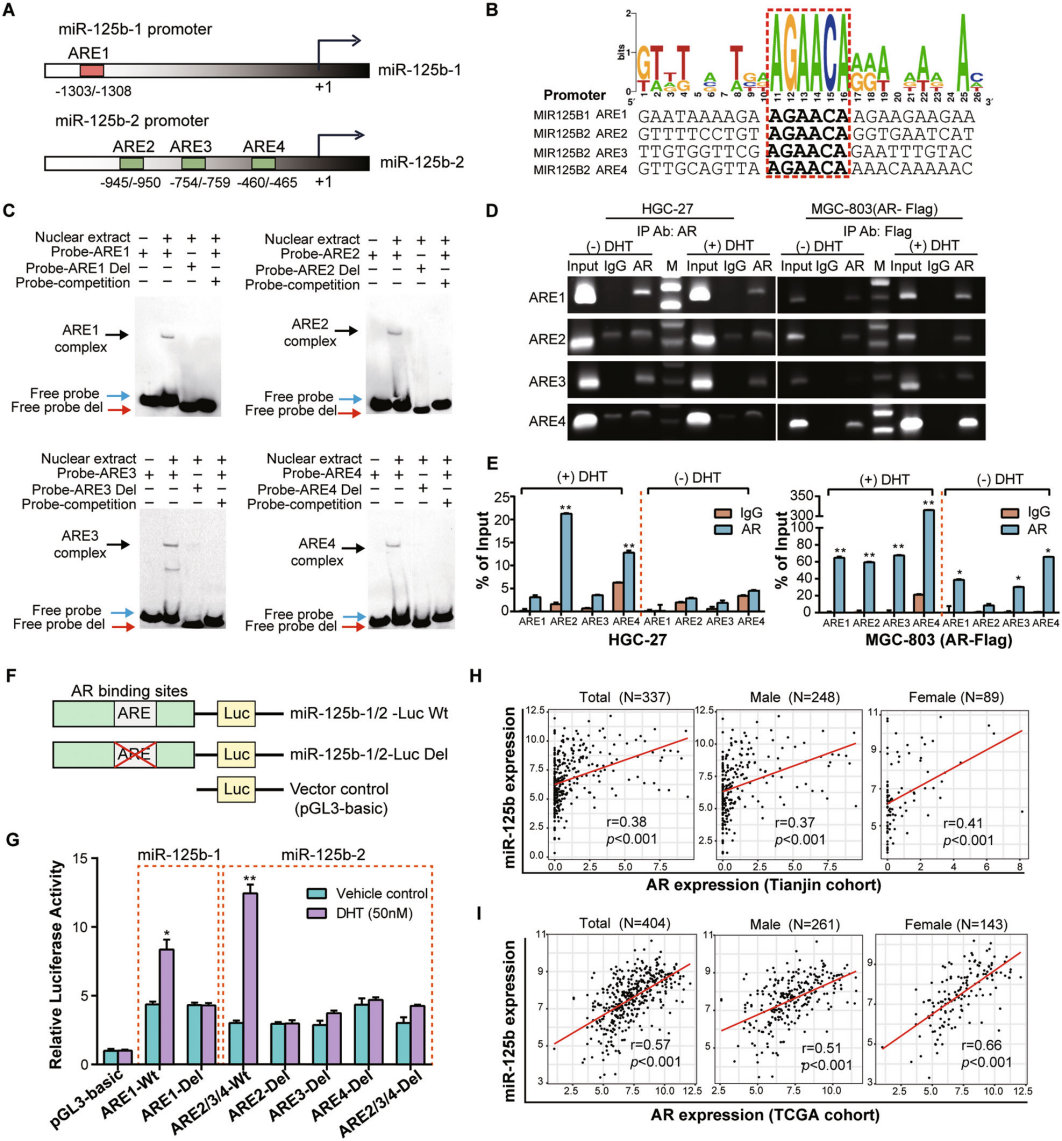
Characterization of AR Binding Sites on the *miR-125b* promoter and the correlation between AR and *miR-125b*. **A** Schematics showing that the human 5′ UTRs of the *miR-125b*-1 and *miR-125b*-2 promoter region contain one (ARE1, -1303/-1308) and three (ARE2, -945/-950; ARE3, -754/-759; ARE4, -460/-465) AREs, respectively. **B** Putative human AREs identified by searching the promoter regions of *miR-125b*-1 and *miR-125b*-2 genes for a motif corresponding to the consensus sequence of ARE. A consensus sequence generated by WEBLOGO displays the blast results of four AREs locations in *miR-125b*-1 and *miR-125b*-2 promotors. **C** Electrophoretic mobility shift assays (EMSA) were performed using nuclear extracts isolated from HGC-27 cells treated with DHT (50 nM) for 24 h. Digoxin-labeled probes span each of the four AR binding sites (ARE1-4) in the miR-125-1 and *miR-125b*-2 promoter. Deleted probes (Probe- ARE1-4 Del) were synthesized with a 6 bp deletion of the ARE on *miR-125b*-1/2 promoters. The specificity of the binding complex was also determined using the unlabeled wild probe as a competitor. The black, blue and red arrows indicate the specific binding complexes for each probe, respectively. **D** ChIP assays performed in HGC-27 and MGC-803 stably expressing FLAG-tagged AR cells treated ±DHT (50 nM) for 24 h. Immunoprecipitation was using the antibodies against AR (for HGC-27), Flag (for MGC-803), or IgG control. Input represents 10% of the total cell extract used for each immunoprecipitation. Lane M: 1 kb DNA marker (**E**) ChIP-qPCR for measuring AR binding sites (ARE1-4) at *miR-125b*-1/2 promoters in HGC-27 cells and MGC-803 transfected with AR-Flag vector after treated with ±DHT (50 nM) for 24 h. Specific PCR primers covering the ARE region of the *miR-125b*-1 or *miR-125b*-2 promoters were used for the PCR analysis, and data were presented as a ratio to the input. **F** Schematic diagram of the construction of luciferase reporter plasmid containing wild type (Wt) ARE and ARE deletion mutants (Del) used in this study. **G** 293-T cells were stimulated with DHT (50 nM) or vehicle control for 24 h after transfection with indicated ARE wild type or deletion plasmids together with a Renilla luciferase reporter gene and incubated for another 24 h. The luciferase activity was measured, and the firefly/renilla ratio was calculated for each dataset. Error bars are means ± SD of three independent experiments. (*P < 0.05, **P < 0.01) (**H**) The correlation between the expression level of AR and *miR-125b* in the tissues of 337 GC cases of the Tianjin cohort (left panel), 248 male cases (middle panel), and 89 female cases (right panel) measured by real-time RT-PCR (TaqMan). **I** The correlation between the expression level of AR and *miR-125b* in the tissues of 404 GC cases of TCGA GC cohort (left panel), 261 male cases middle panel), and 143 female cases (right panel).

To understand the mechanism for *miR-125b* overexpression in GC, we examined the regulatory regions (2 kb upstream of the transcriptional start site) of *miR-125b-1* and *miR-125b*-2. PROMO 3.0 program identified four “AGAACA” androgen responsive elements (AREs) (Fig. 3A, B and Supplementary Fig. S4). These putative AREs were evaluated for their binding to AR through EMSA assay with nuclear extracts obtained from HGC-27 cells treated for 24 h with 5α-dihydrotestosterone (DHT) using a probe including the consensus binding sites for AR (Fig. 3C). The formation of the binding complex was observed in all EMSAs (black arrows). This band was not observed with a probe in which the core six bases of consensus site (red arrows) were deleted or when 200× unlabeled competitors (blue arrows) were added. Subsequently, ChIP assays with HGC-27 and AR stable transfected MGC-803 cells also revealed that AR bound to the *miR-125b* ARE promoter region (Fig. 3D, E). The binding affinity was markedly induced by DHT stimulation determined by ChIP-qPCR (Fig. 3E). Next, we generated reporter gene vectors with the wild type or deletion ARE sequences with the half-site “AGAACA” at *miR-125b*-*1/2* promoter regions in the pGL3-basic vector and transfected them into 293-T cells (pGL-*miR-125b-1* and pGL-*miR-125b-2* including ARE2, ARE3, and ARE4) (Fig. 3F). The luciferase assay showed that both AR binding regions at *miR-125b-1* and *miR-125b*-*2* demonstrate promoter activity with varying degrees of androgen dependence. As expected, the deletion of ARE1 within *miR-125b-1* and any ARE or all three within *miR-125b-2* prominently reduced its promoter activity (Fig. 3G).

A positive correlation was observed between *AR* and *miR-125b* expression among the 337 GC tissues (Pearson *r* = 0.38, *P* < 0.001) (Fig. 3H) and in both male and female GC patients (Pearson *r* = 0.37, *P* < 0.001 for male; Pearson *r* = 0.41, *P* < 0.001 for female). The positive correlation between AR and *miR-125b* were also found in the TCGA cohort of 404 GC cases (Pearson *r* = 0.57, *P* < 0.001) regardless of sex (Pearson *r* = 0.51, *P* < 0.001 for male; Pearson *r* = 0.66, *P* < 0.001 for female respectively) (Fig. 3I).

### *miR-125b* is oncogenic in GC cells

Following our initial report, the expression of *miR-125b* in the 90 GC cases was significantly higher in tumor tissues than in adjacent tissue (*P* = 0.03) (Supplementary Fig. S5A). We performed correlative analysis on the expression levels of *miR-125b* with clinicopathological features in an expanded cohort of 373 GC cases (Supplementary Table S1).

**Fig. 4:**
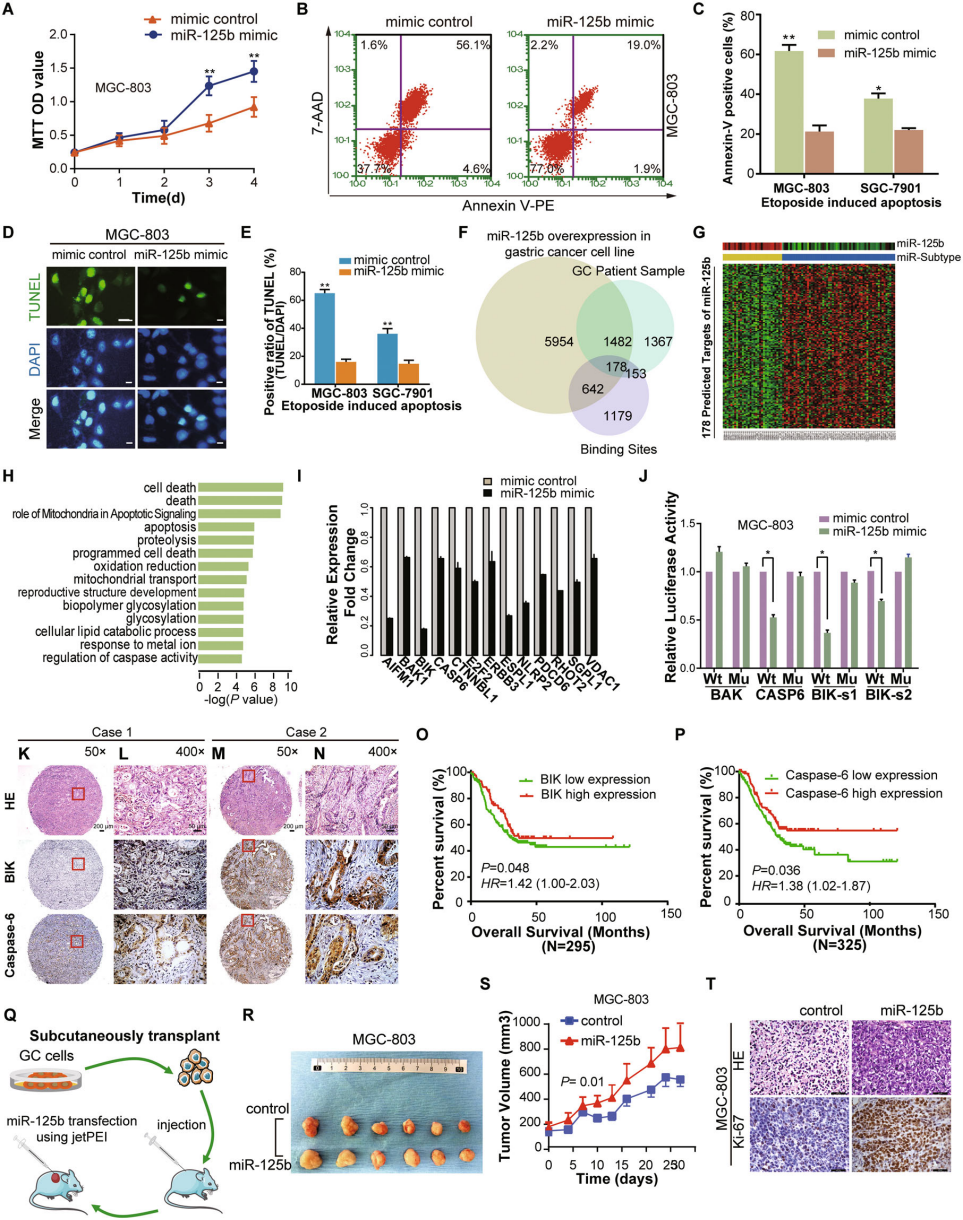
*MiR-125b* suppressed cell apoptosis and promoted proliferation in gastric cancer. **A** MGC-803 cell lines were transfected with *miR-125b* mimic or mimic control, and the MTT assay measured cell proliferation. **B** Representative dot plots of Annexin V/7-AAD staining of MGC-803 cells. After transfected with *miR-125b* mimic or mimic control for 48 h, the GC cells were incubated for 24 h with 40 μM etoposide. Then GC cells were stained with Annexin V/7-AAD dual staining solutions and detected by flow cytometry (FCM). The apoptotic rate was represented as a percentage of total cell populations. The proportion of early apoptotic cells (Annexin V+/7-AAD-, lower right), late apoptotic/necrotic cells (Annexin V + /7-AAD + , upper right), live cells (Annexin V-/7-AAD-, lower left) and dead cells (Annexin V-/7-AAD + , upper left) were respectively measured for comparison. Values in the images indicate the percentage of each fraction. **C** The column bar graph showed the Annexin V positive apoptotic cells of flow cytometry data presented in MGC-803 and SGC-7901 cells. The data are presented as mean ± SD of three independent experiments. *P < 0.05 **P < 0.01 indicates a significant difference as compared to the miRNA mimic control group. **D** and **E** Identification of late apoptosis by fluorescent TUNEL assay after transfection of *miR-125b* and mimic control for 48 h and incubated with 40 μM etoposide for 24 h. The stained cells were observed under a fluorescence microscope. The representative images were shown in (**E**). The Positive cells show a bright green nucleus and DAPI staining was used to show the location of the nucleus. Columns in (**E**) represent the TUNEL positive cell number in *miR-125b* and control group. Scale bar = 20 µm (**F**) A Venn diagram shows the overlap results of integrated analysis dataset (3′-UTR Binding site searching, *miR-125b* transfected cell line and patient sample dataset), which showed 178 targets of *miR-125b*. **G** Heatmap shows the expression of 178 targets. miR-subtype and *miR-125b*’s expression are shown at the top of the heatmap. **H** Pathway analysis shows that the predicted targets of *miR-125b* are highly involved in the apoptosis/program death pathway. **I** Representative apoptosis genes (e.g., BIK, BAK1, CASP6) are all downregulated after *miR-125b* transfection. **J** Effect of *miR-125b* overexpression on a dual-luciferase reporter plasmid containing the 3′-UTR of candidate target genes (BAK, CASP6, and BIK) was analyzed. The GC cells were co-transfected with either the wild type pMIR-Wt-3′-UTR- BAK/CASP6/BIK (Wt) or corresponding mutant 3′-UTR (Mu) or an empty vector and *miR-125b* or mimic-control. Firefly and renilla luciferases were measured in the MGC-803 cell lysate. **K–N** BIK and Caspase-6 protein expression measured by immunohistochemical and HE staining in a tissue microarray (TMA). Immunohistochemical analysis on consecutive tissue microarray slides of GC tissues showed different expression of BIK and Caspase-6 in GC patient. Representative case 1 had low expression of BIK and Caspase-6 (K and L, respectively). Representative Case 2 represented a high expression of BIK and Caspase-6 (**M** and **N**, respectively). Scale bars, 200 μm (**K** and **M**) and 50 μm (**L** and **N**), respectively. **O–P** Kaplan–Meier overall survival curves according to low and high BIK (**O**) and Caspase-6 (**P**) protein expression in 295 and 325 cases, respectively. Green and red lines represent low and high protein expression, respectively. **Q** Schematic representation of *miR-125b* transfection experiment using xenografted MGC-803 and SGC-7901 nude mice model. **R** Representative images of tumor sizes in control and miR-125b treated mice from MGC-803 cells. **S** In vivo xenograft tumor growth curve of MGC-803 cells expressing control and miR-125, error bars represent ± SEM. **T** the representative images of HE and Ki-67 staining of MGC-803 xenograft tumors after 4 weeks of *miR-125b* and control in vivo transfection. Scale bar = 50 µm.

Compared to the control, the number of viable cells was increased over time in both cell lines (MGC-803 and SGC-7901) transfected with *miR-125b* mimic (*P* < 0.05) (Fig. 4A and Supplementary Fig. S5B). The Annexin V-PE/7-AAD double staining method was used to detect etoposide (40 μM)-induced apoptosis. The result showed that *miR-125b* overexpression inhibited apoptosis (Fig. 4B, C and Supplementary Fig. S5C). Consistently, *miR-125b* overexpression decreased the late apoptosis of GC cells measured by TUNEL assay in the same treated cells as described in Fig. 4A and Supplementary Fig. S5B (*P* < 0.05) (Fig. 4D, E and Supplementary Fig. S5D).

### The predicted target genes of *miR-125b* are involved in apoptosis/program death pathway

To further explore the target genes and pathway of *miR-125b* in GC, we performed an integrated analysis of comprehensive data including the 3′-UTR binding site searching with Targetscan database (http://www.targetscan.org), transcriptome profiles from *miR-125b* transfected cell line (accession number GSE145959) and the previous Tianjin GC data^29^ (Fig. 4F). We predicted 178 targets of *miR-125b* whose expression was inversely correlated with *miR-125b* expression (Fig. 4G and Supplementary Table S4). The predicted targets of *miR-125b* are enriched in the apoptosis/program cell death pathway (Fig. 4H and Supplementary Table S5). Microarray analysis showed that the representative apoptosis genes (e.g., *BIK, BAK1*, and *CASP6*) were all downregulated after *miR-125b* transfection (Fig. 4I). Using the target prediction tool (TargetScan database), we identified a putative *miR-125b*-binding site located in the 3′-UTR of the three apoptosis genes (Supplementary Fig. S5E). *miR-125b* significantly suppressed the luciferase activity in the BIK and CASP6 but not BAK compared to *mimic-control* in cells transfected with wild-type vectors but not in the mutant one (Fig. 4J and Supplementary Fig. S5F-H), suggesting that *miR-125b* directly binds to the 3′-UTR of *BIK* and *CASP6* mRNA. Overexpression of *miR-125b* can downregulate mRNA and protein levels of *BIK* and *CASP6* (Supplementary Fig. S5I, L).

Compared to high expression, low BIK and Caspase-6 expression (Fig. 4K-N) were significantly associated with poor OS (*P* = 0.048 and 0.06 respectively) and unfavorable DFS (*P* = 0.036 and 0.03 respectively) (Fig. 4o, p and Supplementary Fig. S5M, N). Cox regression analysis confirmed that Caspase-6 remained statistically significant for shorter OS after adjusting for confounding variables (*HR* = 0.72; *P* = 0.033), while a borderline correlation was observed between lower BIK expression and poorer outcome (*HR* = 0.70; *P* = 0.051) (Supplementary Table S3). Statistically significant associations were also found between BIK/Caspase-6 expression and clinicopathological features of GC (Supplementary Table S6).

We established subcutaneous xenograft nude mouse models using two GC cell lines, MGC-803 and SGC-7901, as previously described^29^. The *miR-125b* mimic was delivered by intratumoral injection in the model mice using in vivo JetPEI (Polyplus-Transfection) as a carrier (Fig. 4Q). *miR-125b* overexpression significantly enhanced the growth of tumor xenografts from both MGC-803 (*P* < 0.05, Fig. 4R, S) and SGC-7901 (*P* < 0.05, Supplementary Fig. S6A, B) cells. Proliferative activity assessed using the Ki-67 immunohistochemical (IHC) staining was higher in the group of *miR-125b* overexpression, compared to that of the miRNA control groups (*P* < 0.05, Fig. 4T and Supplementary Fig. S6C-E).

### The AR antagonist, bicalutamide, inhibits GC cells in vitro and in vivo

**Fig. 5:**
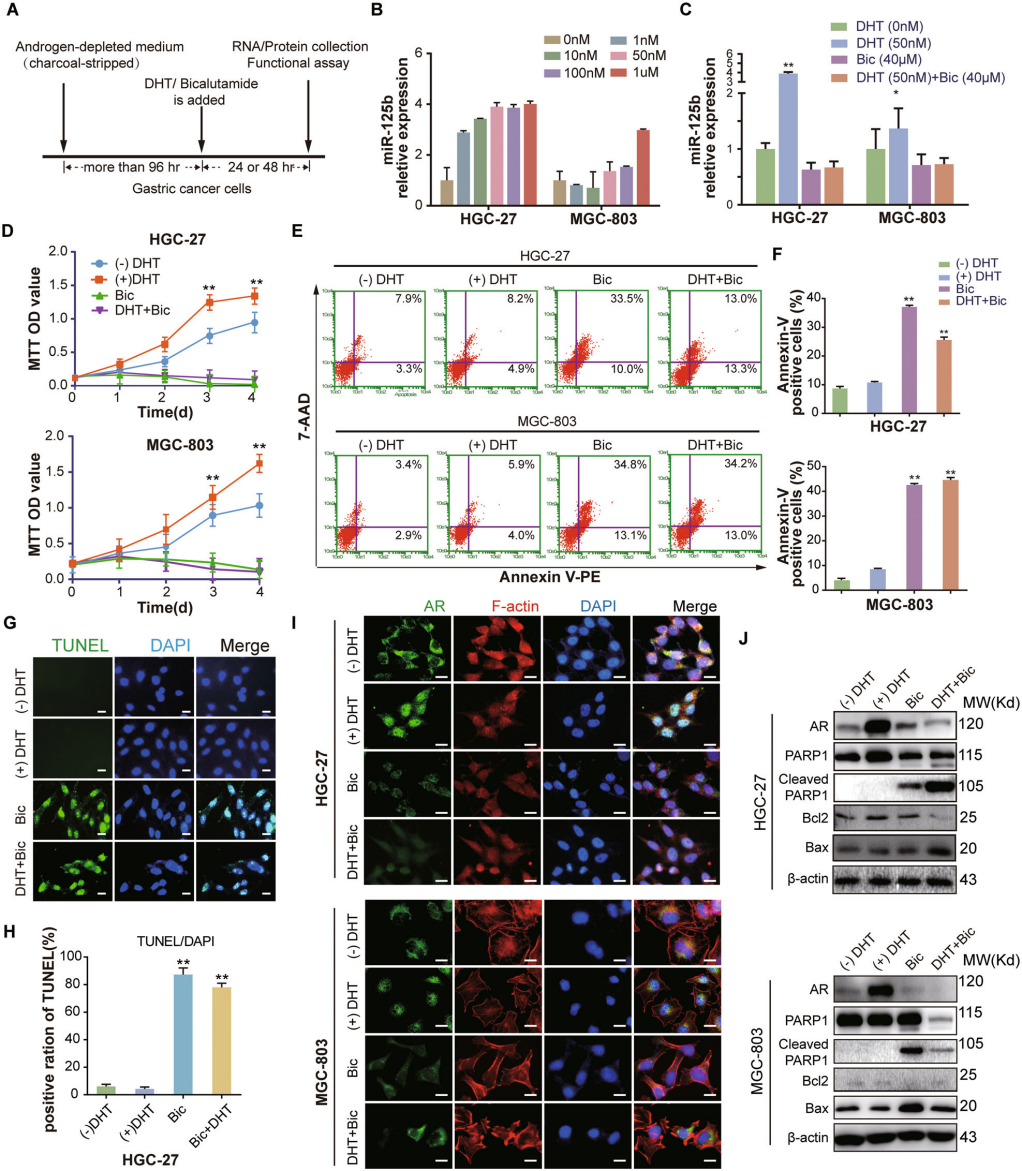
The effect of bicalutamide as AR antagonist on proliferation and apoptosis in GC cells. **A** Schematic experimental workflow for the treatment of GC with DHT and/or bicalutamide (BIC). **B** HGC-27 and MGC-803 cells were cultured in the androgen-depleted medium for 4 days and then treated with vehicle ((-) DHT) or 1, 10, 50, or 100 nM DHT for 24 h. RT-qPCR was used to measure the level of *miR-125b* expression in GC cells. **C** HGC-27 and MGC-803 cells were treated with vehicle control, DHT (50 nM), and bicalutamide (BIC, 40 μM) with or without DHT for 24 h. The level of *miR-125b* expression in GC cells was measured by RT-qPCR. **D** MTT assays were used to measure HGC-27 (upper panel) and MGC-803 cell (lower panel) proliferation in the four experimental groups mentioned in Fig. 6c. **E** Representative dot plots of Annexin V/7-AAD dual staining to detect the effect of Bicalutamide on apoptosis. The HGC-27 (upper panel) and MGC-803 (lower panel) cells treated with DHT and/or BIC mentioned in Fig. 6c for 24 h. Then GC cells were stained by flow cytometry (FCM). Apoptotic cell rate was represented as a percentage of total cell populations, as mentioned in Fig. 2b. **F** The column bar graph showed the Annexin V positive apoptotic cells of flow cytometry data presented in (**E**). The data are presented as mean ± SD of three independent experiments. *P < 0.05 **P < 0.01. **G** and **H** Identification of late apoptosis by fluorescent TUNEL assay after treatment with Bic and/or DHT for 48 h in HGC-27 cells. Columns in (**H**) represent the TUNEL positive cell numbers of all treatment groups and vehicle control group in (**G**). Scale bar = 20 µm (**I**) Immunofluorescence analysis of AR nuclear translocation in HGC-27 (upper panel) and MGC-803 (lower panel) cells treated with Bic (40 µM) and/or DHT (50 nM) for 24 h. Green fluorescent: AR, the red: F-actin, the blue: DAPI staining of nuclear. Scale bar = 20 µm. **J** Immunoblot of AR, PARP1, cleaved PARP1, Bcl-2 and Bax in HGC-27 (upper panel) and MGC-803 (lower panel) cells described above (**C**) in the presence or absence of DHT and/or bicalutamide. β-actin: internal reference.

We next sought to test whether AR inhibitors have therapeutic value in GC by blocking the AR/*miR-125b* axis (Fig. 5A). We conducted a series of in vitro and in vivo experiments by using bicalutamide, a clinical AR antagonist. AR was observed in HGC-27, MGC-803, and SGC-7901 cell lines, but not in BGC-823, NCI-N87, and AGS cell lines (Supplementary Fig. S7A, B). Treatment of both HGC-27 and MGC-803 cells using androgen (DHT, 1.0–1 μM) for 24 h markedly increased *miR-125b* expression level in a dose-dependent manner (Fig. 5B). The treatment by bicalutamide (40 μM), single or in combination with DHT (50 nM), decreased the miR-125 level compared to the control groups with or without DHT (Fig. 5C). Bicalutamide treatment suppressed cellular viability measured by MTT assay (Fig. 5D). It elevated both early apoptosis measured by Annexin V/7-AAD dual staining and flow cytometry (Fig. 5E, F) and late apoptosis measured by TUNEL assay in HGC-27 (Fig. 5G, H) and MGC-803 (Supplementary Fig. S7C, D) cells (*P* < 0.05, *t*-test).

The nuclear translocation of AR was detected after the treatment with bicalutamide and/or DHT in GC cells using an immunofluorescence staining assay (Fig. 5I). Specifically, the presence of DHT resulted in a bright AR signal (green fluorescence) in the nucleus while bicalutamide (with or without DHT) markedly reduced DHT-dependent AR nuclear signals and led to diffused cytoplasmic staining.

**Fig. 6:**
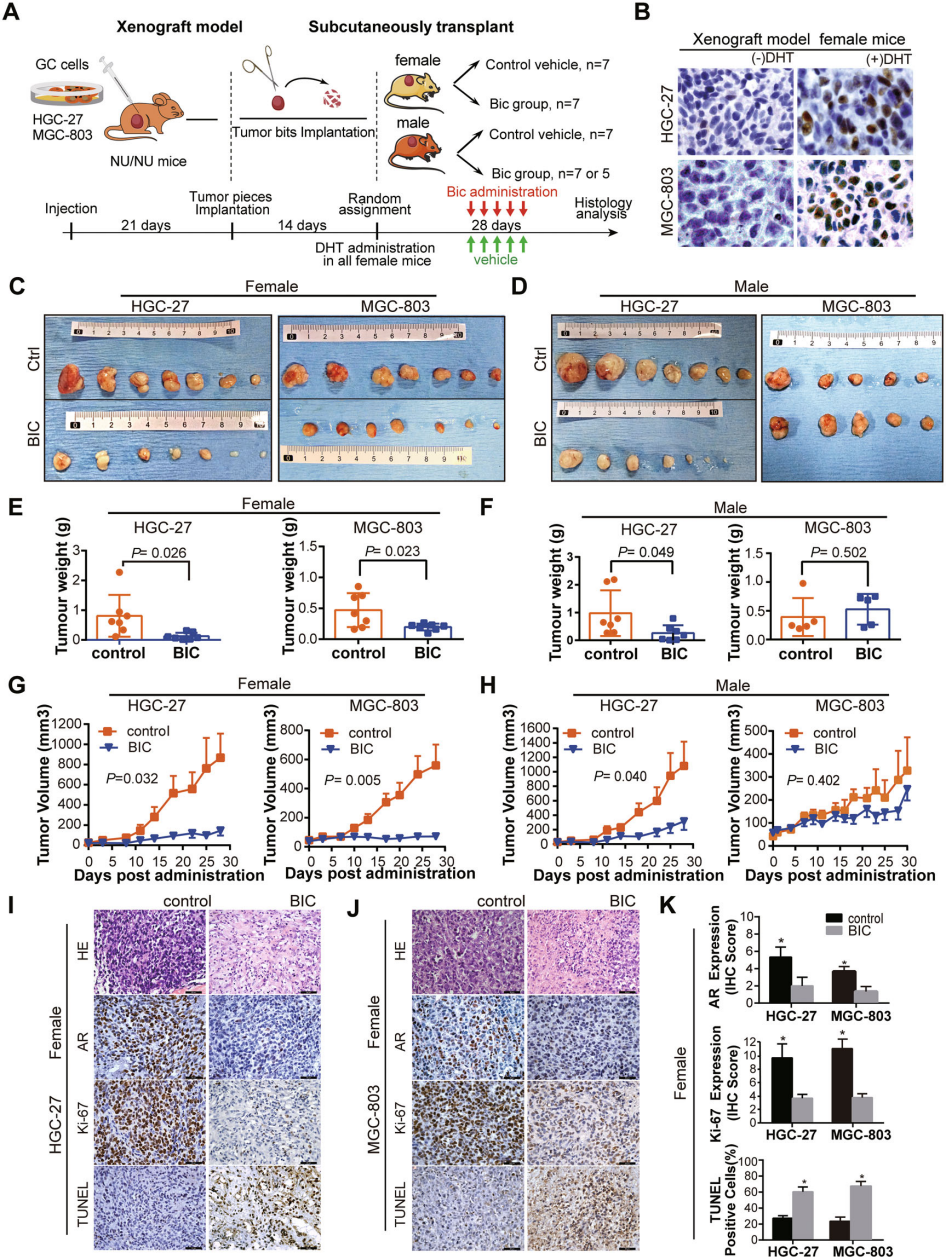
Bicalutamide (Bic) decreases cellular viability and increases necrosis and apoptosis in HGC-27 and MGC-803 xenografts. **A** The flowchart showed the Xenograft model procedure for AR agnostic efficiency test. **B** Two weeks after inoculation, The AR protein expression of MGC-803 tumor samples from control (upper) and DHT (lower) treated mice were evaluated by immunohistochemistry (IHC). Scale bars represent 50 μm. **C** and **D** Representative images of tumor nodules in control (ctrl) and BIC (50 mg/Kg/Day) group of female (**C**) and male (**D**) xenograft model. Note: HGC-27 (left panel) and MGC-803 (right panel) xenografts were displayed respectively. Scale bar represents 1 cm. **E** and **F** Quantification of tumor nodule weights in control and BIC administrate group of mice of HGC-27 (left) and MGC-803 (right) in female (**E**) and male (**F**) xenograft model, error bars represent ±SD. **G** and **H** The growth curve of tumor xenografts in nude mice of HGC-27 (left) and MGC-803 (right) in female (**G**) and male (**H**) xenograft model, error bars represent ± SEM. **I** and **J** The AR and Ki-67 protein expression and apoptosis status of HGC-27(**I**) and MGC-803(**J**) in female xenograft model, tumor samples from control and BIC treated mice were evaluated by H&E staining, immunohistochemistry (IHC), and TUNEL assay. Scale bars represent 50 μm. **K** Quantification of AR and Ki-67 protein expression and TUNEL positive cells in HGC-27 (**I**) and MGC-803 (**J**) tumor samples of female xenograft model. Error bars represent ± SD. *Indicates significant difference *P* < 0.05.

The above in vitro experiments showed that inhibition of AR might provide therapeutic value in GC. We thus performed preclinical mouse model experiments to examine the effect of AR drug on AR-positive GC cell line HGC-27 and MGC-803 (Fig. 6). We first used female mice considering the association of AR with poor prognosis only in female GC patients (Fig. 1H). In a preliminary experiment on female BALB/c nude mice with MGC-803 and HGC-27 cell xenografts, DHT dramatically induced the AR expression of both xenografts in female mice (Fig. 6B). Given the low levels of androgen in female mice, DHT was subsequently given to the host female mice in the following preclinical experiments.

BIC was administrated to mice at 50 mg/Kg once daily (Q.D.). After 4 weeks, the mice were euthanized, and subcutaneous tumors were removed for analysis (Fig. 6A). Representative images from the analyzed tumor sections and the subcutaneous tumors are shown in Fig. 6C, D.

In female mice, the drug administration resulted in a significant reduction in the volume and weight of the tumor compared with the control group in female host mice implanted with either HGC-27 (*P* = 0.026, Fig. 6C, E, left panel) or MGC-803 cells (*P* = 0.023, Fig. 6C, E, right panel). BIC treatment reduced the tumor growth rate in both HGC-27 and MGC-803 female xenograft nude mice (*P* = 0.032 and 0.005, respectively, Fig. 6G). The results confirmed that BIC decreased the AR expression and inhibited tumor proliferation in both female host mice (Fig. 6I-K). Concomitantly, apoptosis was induced in the BIC treatment group measured by TUNEL assay (Fig. 6I-K).

However, in male mice, BIC inhibited tumor growth only in HGC-27 but not MGC-803 xenografts (Fig. 6D, F, H). Correspondingly, the growth curve showed that BIC significantly inhibited the growth of GC cell derived xenografts (*P* = 0.040 and *P* = 0.402, respectively, Fig. 6H). Consistently, BIC administration inhibited the proliferation and apoptosis of HGC-27 but not MGC-803 xenografts (Supplementary Fig. S8A, B).

### The potential anti-tumor activity of bicalutamide was accessed by using the preclinical PDX mice model

**Fig. 7:**
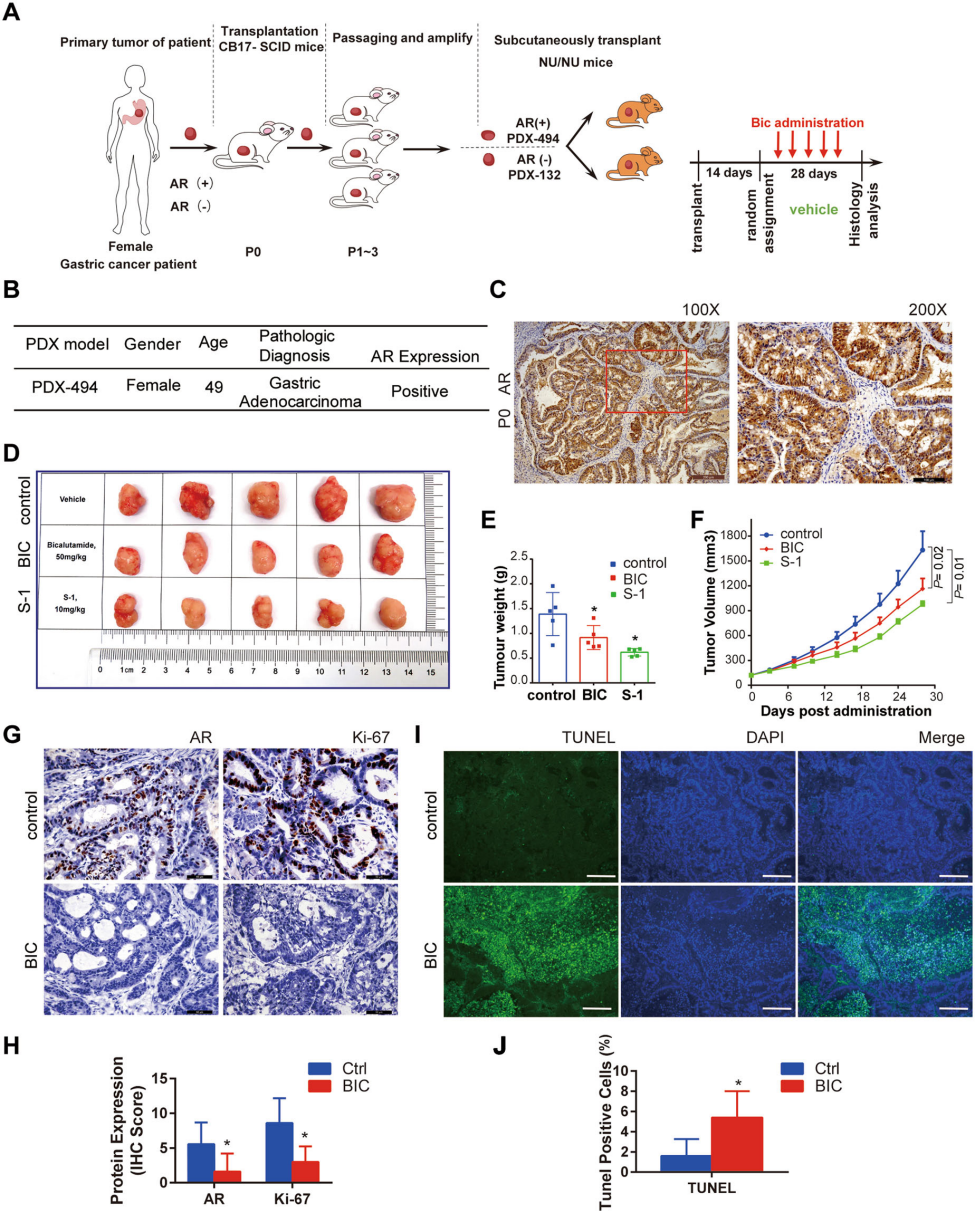
In vivo anti-tumor activity of Bicalutamide (Bic) were detected in PDX-494 mice model from AR-positive female GC patient. **A** The schematic representation of the generation of the female GC PDX tumor model procedure for AR agnostic efficiency test. **B** Patient Information of PDX-494 is shown in the table. **C** AR expression in P1 (the first passage) of PDX-494 was measured by immunohistochemical staining. Scale bars, 200 μm (left panel) and 100 μm (right panel), respectively. **D** Representative images of tumor nodules in control, BIC (50 mg/Kg/Day), and positive control S-1 group of PDX model. Scale bar represents 1 cm. **E** Quantification of tumor nodule weights in control, BIC, and S-1 administrate group of PDX model. Error bars represent ± SD. **F** The growth curve of tumor xenografts of control, BIC, and S-1 administrate group in the PDX model, error bars represent ± SEM. **G** The AR and Ki-67 protein expression in PDX model, tumor samples from control and BIC treated mice were evaluated by immunohistochemistry (IHC). Scale bars represent 50 μm. **H** Quantification of AR and Ki-67 protein expression in PDX-494 model. **I** Apoptotic cells were detected using the TUNEL immunofluorescence technique. Scale bars represent 50 μm. **J** Quantification of apoptosis status in PDX-494 model from control and BIC treated mice was evaluated by TUNEL assay.

We further evaluated the anti-tumor activity of bicalutamide in the female GC patient-derived xenograft (PDX) model (Fig. 7 and Supplementary Fig. S9). Two female patient-derived tumors, with AR-positive (PDX-494, Fig. 7B, C) and AR- negative (PDX-132, Supplementary Fig. S9A, B) expression, were adopted in this preclinical investigation. As described above, the nude mice bearing xenografts of PDX tumor cells were treated with 50 mg/Kg BIC once daily (Q.D.). S-1, a common 5-fluorouracil (5-FU)-based chemotherapy regimens, was administrated to mice as a positive control of drug efficiency (Fig. 7A). For the reasons mentioned above (Fig. 6B), we use the male mice in all PDX tumor experiments to ensure sufficient expression of androgen and AR activation.

In the PDX-494 model, BIC treatment significantly inhibited tumor growth with reduced tumor volumes (Fig. 7D, E) and suppressed growth curve (*P* = 0.02) (Fig. 7F) compared to the control group. BIC administration can inhibit the proliferation (Fig. 7G, H) and apoptosis of PDX-494 xenografts (Fig. 7I, J).

However, for the results of PDX-132 model, there was no significant difference in the tumor growth between the two groups (control, BIC) (Supplementary Fig. S9C-E). Consistent with our expectation, the results were also confirmed by IHC (AR and Ki-67) and TUNEL staining (Supplementary Fig. S9F-I).

## Discussion

**Fig. 8:**
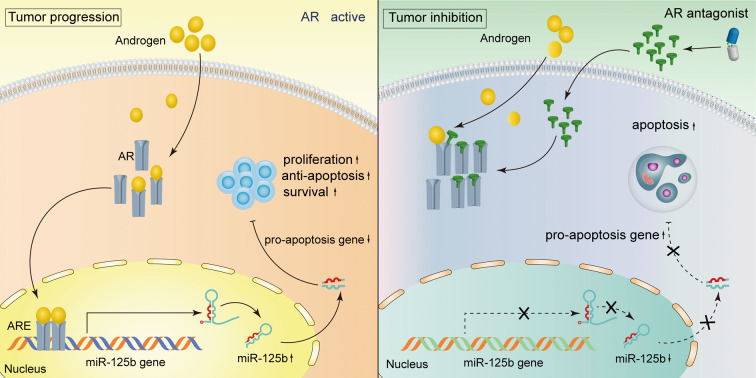
Schematic proposed model of AR/*miR-125b* regulatory circuit that controls gastric cancer development and progression. Left panel: AR active was shown when adding androgens and entry nucleus as a transcription factor. Then AR can bind the AREs in the promoter of the *miR-125b* gene and up-regulate the *miR-125b* expression. The *miR-125b* targeted pro-apoptosis genes are down-regulated and induce gastric cancer cell growth and progression. Right panel: when the AR antagonist competitive binding AR and excluded AR from the nucleus. The AR/*miR-125b* circuit is coordinately inhibited and favors the apoptosis pathway activation under these conditions. The growth of GC cells is inhibited. Discontinuous arrows represent the inhibition, consecutive represent stimulation or release. AR androgen receptor, ARE androgen response element.

In this study, we first explored extensive gender-biased gene signatures in multiple large GC datasets that include a total of 1390 GC cases by deep learning and gene enrichment analysis. The sex chromosome abnormalities and AR-related pathway deregulation may significantly contribute to gender disparities. These studies have revealed a hormone-dependent regulatory circuit in GC development and progression (Fig. 8).

The most important finding in the current study was many X-chromosome genes (such as XIST, KDM6A, ZFX, and KDM5C), known to escape X-chromosome inactivation^30^, strongly contributed to the gender difference in GC. These results corroborate the previous findings that females would be protected by the subsets of putative tumor-suppressor genes that can escape this silencing and be expressed from both X chromosomes in females^31–33^. These results plausibly explained the dilemma that males tend to present with more aggressive cancers and higher mortality than females. Another most noteworthy is that the X-chromosome contains an excess of genes related to hormones and hormone receptors such as AR. Our results support the hypothesis that X-chromosome deregulation may affect the AR function and have relevance to female carcinogenesis (i.e., ovarian and breast cancer)^34,35^. Moreover, the modulation process of AR by sex chromosomal abnormalities is age-related and cell type-specific^36^, which may lead to the gender differences in GC.

The effect of AR or *miR-125b* on the poor prognosis of female patients suggested that the male hormone, when abnormally disturbed in female cancer cells, has a more deleterious impact on the clinical outcome. In other male-dominant cancer, such as hepatocellular carcinoma or bladder cancer, AR and androgen are reported to involve in carcinogenesis and associate with worse outcomes^37–39^. Correspondingly, in preclinical mouse model studies, the anti-tumor activity of the AR inhibitor was more marked in female mice harboring GC cells. More interestingly, in male mice, the drug has more effective in these animal models of HGC-27 than in MGC-803 cells, which may attribute partly to the different AR levels in these two GC cell lines. Another factor that is worth considering is HGC-27 cells derived from female, MGC-803 derived from male. These results showed that GC in females may be more sensitive to androgen than males. In vivo experiments confirmed gender differences in certain chemical carcinogens induced bladder cancer mouse model^40^. Based on all these epidemiologic and experimental observations, we hypothesized AR signals are a potential mediator of gender-specific differences in GC.

The reported roles of *miR-125b* have exhibited cancer type-dependent variances. The present study consistently supports an oncogenic role of *miR-125b* in GC^41–43^. The up-regulation of *miR-125b* was also reported in hematological malignancies, and other solid tumor types^27,44–46^. On the other hand, decreased expression in *miR-125b* has been reported in liver cancer^47,48^, and breast cancer^49^. Thus, *miR-125b* may be one of the miRNAs that may have pleiotropic functions as either oncomiR or tumor suppressor, depending on the tissue context and regulatory environment.

To our best knowledge, AR inhibitor has not been tested in the clinical treatment of GC. Promising AR-targeted strategies in clinical trials have been reported in several non-prostatic cancers, such as hepatocellular carcinoma, breast cancer, bladder cancer, and ovarian cancer^37,50–53^. The role of the AR/*miR-125b* axis in GC revealed by our study provides the initial preclinical evidence to support the AR-targeting clinical trial in GC. Further translational studies are warranted to pursue gender disparity and cancer therapy in GC.

## Materials and methods

### Experimental design

The study was approved by the Institutional Review Board of Tianjin Medical University. Gastric cancer (GC) specimens and clinical information were obtained from Tianjin Medical University Cancer Institute and Hospital with informed consent from all patients. All samples were made anonymous and handled according to ethical and legal standards. Our present study included a discovery phase and a validation phase. In the discovery phase, we constructed a dataset with genome-wide microRNA and mRNA expression profiling of 90 Tianjin GC samples^29^. Another four GC datasets in the present study were obtained from public GC databases, including TCGA, ACRG, and GEO databases (GSE15459 and GSE84437). In the validation phase, we performed validation of candidate genes. We evaluated their potential as biomarkers of GC survival in 373 GC cases in Tianjin, including the 90 cases in the discovery phase. A total of 373 patients included in this study was histologically confirmed new cases of GC. The demographic and clinical characteristics of patients were summarized (Supplementary Table S1), and the patients were followed up from surgery in January 2016 through regular telephone contacts and clinical visits. Survival time was calculated from the date of diagnosis to the date of death or the last follow-up date.

### mRNA and MicroRNA expression profiling

We acquired the mRNA expression profile using Affymetrix HG-U133 Plus 2.0 arrays (Affymetrix, Santa Clara, CA) and miRNA expression profiles using GeneChip^®^ miRNA Arrays (Affymetrix). Microarray data processing and Statistical analysis were performed as previously described^29^. The lncRNA IDs from HG-U133 Plus 2.0 array were re-annotated using the biomaRt R package.

### Differential gene expression analysis with deep learning

In this study, we developed a deep neural network of 11 layers with dense connection^54^ to predict the sex status (i.e., male versus female) of an individual by taking input as a gene expression profile. We employed an integrated gradient algorithm^55^ to identify genes that are associated with sex disparity. The integrated gradient is a new attribution method that can attribute the prediction of a deep learning model to its input features. We then calculated the attribution score of each gene. The average attribution score of a given gene in the male group reflected the association of this gene with sex disparity. The reads count gene expression data of TCGA was prepossessed by cpm function in edgeR package^56^. Subsequently, we used the scale function in R software to normalize the expression matrix by subtracting features by their means and dividing by their standard deviations. We randomly selected 10% of samples as a validation set and the rest as a training set. We trained the deep neural network iteratively for 200 epochs. The validation set was used to select the best model among these 200 models. We used the best model and the aforementioned integrated gradient algorithm to calculate the attribution score of each gene in the TCGA cohort. The attribution scores were used as statistics to run gene set enrichment analysis (GSEA). GSEA was conducted with R package fgsea. The Venn diagram and the pathway heatmap were generated using the TBtools (version 0.674)^57^.

### Differential gene expression (DGEs) and gene enrichment analysis

We divided patients into two groups according to gender or AR expression level in Tianjin and TCGA dataset, respectively. Then we calculated the difference between groups using linear models (Limma package) and screened out the genes with a significant difference according to *P* value < 0.05 and Foldchange > 0.5. The selected DGEs of lncRNA and miRNA were used to perform Metascape Functional enrichment analysis and generate heatmaps.

Gene enrichment analysis was performed with an online analytical tool Metascape with custom analysis settings (http://metascape.org/gp/index.html)^58^. The bar plot, heatmaps, and circos plot were used to describe the enrichment results and visualized using the TBtools^57^

### TCGA data and GEO resource

The Cancer Genome Atlas (TCGA) mRNA and microRNA expression data with clinical information of GC patients were downloaded from Genome Data Commons (https://portal.gdc.cancer.gov) and UCSC Xena (https://xenabrowser.net/). The expression data were normalized by log2 transformation using the preprocessCore in the R/Bioconductor package. The public GC datasets of GSE66229 for ACRG, GSE15459 and GSE84437 were obtained from GEO database (http://www.ncbi.nlm.nih.gov/geo/).

### Promoter activity assays

We generated luciferase reporter constructs by inserting PCR amplified fragments of upstream of *miR-125b-*1 (963 bp) and *miR-125b-*2 (745 bp) TSS into the Kpn I and Mlu I sites of pGL3-Basic. All ARE deletions of *miR-125b* promoter described here were created using the PCR-based mutagenesis method and confirmed by DNA sequencing. 293T cells were transfected with 300 ng luciferase reporter plasmids and 5 ng TK (thymidine kinase) control plasmid with Lipofectamine 3000. In accordance with the routine culture, the medium was changed after 8 h and DTH was added. Luciferase activity was examined 24 h post-transfection by the Dual-Glo Luciferase Assay System (Promega, Madison WI) and recorded by a GloMax 20/20 (Promega, USA). The results were displayed as the mean ± SD of three independent experiments, each performed in triplicate. Data were analyzed with GraphPad Prism (Intuitive Software for Science, San Diego, CA). Statistical significance was determined using an ANOVA one-way test, and differences were considered significant at a *P* < 0.05.

### miRNA binding activity assays

For analysis of predicted *miR-125b* binding sites, synthesize complementary 50–60 mer DNA oligonucleotides consisting of the 3′-UTR sequence of candidate target genes that were cloned into pmirGLO Dual-Luciferase miRNA Target Expression Vector (Invitrogen, USA). The blank vector was used as a negative control. The sequences of oligonucleotide pairs that contain the desired miRNA target region are shown (Supplementary Table S8). The Design oligonucleotides are cloned into pmirGLO vectors according to the manufacturer’s instructions. MGC-803 and SGC-7901 cells maintained in the 24-well plates were transfected with 0.4 µg of the pMIR-REPORT Luciferase plasmids (Wild type and mutant vectors) and miRNA (*miR-125b*/mimic control) using the Lipofectamine 3000 (Invitrogen, USA). The relative luminescence was measured using the Dual-Luciferase Reporter Assay Kit (Promega, USA) 24 h post-transfection.

### Annexin V apoptosis assay

The Nexin assay was performed according to the manufacturer’s protocol. Briefly, the GC cells were seeded in a 12-well plate and incubated with apoptosis inducer or bicalutamin/for 12 or 24 h. The adherent cells were harvested by trypsinization and pelleted by refrigerated centrifugation at 300 *g* for 10 min. Re-suspended cells in 100 μL medium with 10% FBS were mixed with 100 μL, the Guava Nexin^TM^ reagent containing Annexin V-PE and Nexin 7-amino-actinomycin D (7-AAD), loaded onto the 96-well plate and incubated at room temperature in the dark for 15 min. Samples containing 5 × 10^3^ cells were analyzed by using a Guava EasyCyte flow cytometer (Guava Technologies, Hayward, CA, USA) with the guavaSoft 3.3. This assay can detect the exposure of phosphatidylserine (PS) on the cell surface, which is the early changes in cell membrane structure in apoptosis. Annexin V is a phospholipid-binding protein that has a high affinity for PS translocated to the cell surface. 7-AAD is a cell impermeant dye excluded from live cells but taken up by late-stage apoptotic cells as the membrane becomes porous. Cells that stain positive for both dyes are in the later stages of apoptosis. Cells were gated based on forward scatter (size), and results are reported as the percentage of gated cells that are positive for both annexin V and 7-AAD.

### TUNEL (Terminal deoxynucleotidyl transferase-mediated dUTP nick end labeling) assay for in vitro cell culture

GC cells were glass cover-slipped in 24-well dishes containing growth medium. After 60–70% cell confluence was achieved, the cells were exposed to different treatments. Then the cells were incubated with 50 µL of TUNEL reaction mixture at 37 °C for 1 h in a humid atmosphere. The cells were then stained with 5 µL Bright green labeling mix for 5 min, and the stained cells were visualized under a Leica fluorescence microscope DM2000 (Wetzlar, Germany) a magnification of ×200.

### Chromatin immunoprecipitation (ChIP)

MGC-803 cells overexpressing AR-Flag and HGC-27 cells were crosslinked with 1% formaldehyde at 37 °C for 10 min, rinsed twice with ice-cold PBS, and harvested in PBS containing 1× protease inhibitor cocktail (Thermo Scientific, Waltham, USA). Then harvested cells were centrifuged for 5 min at 3000 × *g*. Break the membrane with Membrane Extraction Buffer containing protease inhibitors cocktail, re-suspend nuclei in MNase Digestion Buffer Working Solution and add MNase to digest the chromatin for 15 min in a 37 °C water bath mixing by inversion every 5 min. Centrifuge at 9000 × *g* for 5 min to recover the nuclei and remove the supernatant. Resuspend nuclei in 100 μL of 1×IP Dilution Buffer containing protease/phosphatase inhibitors and sonicate on ice with several pulses to break the nuclear membrane. Supernatants were collected and diluted in IP dilution buffer. 10 μg antibody was prebound for 6 h to ChIP Grade Protein A/G Magnetic Beads (Thermo Scientific, CA, USA) and then added to the diluted chromatin following by overnight immunoprecipitation. The magnetic bead-chromatin complexes were collected and washed three times in IP Wash Buffer 1 and twice with IP Wash Buffer 2. To reverse the cross-linking, we incubated the magnetic bead complexes for 40 min at 65 °C in IP Elution Buffer. DNA fragments were purified using a DNA Recovery Kit (Thermo Fisher, Waltham, USA). For PCR/qPCR, 1.0 µL from a 150 µL immunoprecipitated chromatin extraction and 40 cycles of amplification were used. Antibodies used were ChIP grade anti-AR (CST, MA, USA), anti-Flag (MBL International, Woburn, MA, USA), and control IgG (Supplementary Table S7). The primers for ChIP qPCR are listed (Supplementary Table S8).

### Chromosomal location and gene structure

Sex chromosome distribution diagrams of all gender-specific genes were drawn by the software MapGene2Chrome V2 (http://mg2c.iask.in/mg2c_v2.1/). The gene structure was drawn using the online tool- Illustrator for Biological Sequences (IBS) (http://ibs.biocuckoo.org/).

### Cell culture, treatment, and transfection

The human GC cell lines MGC-803, HGC-27, SGC-7901, MKN-45, AGS NCI-N87, and BGC-23 were obtained from American type culture collection or the cell bank in the Chinese Academy of Sciences (Shanghai, China). The cell lines in this study were authenticated by the STR profiling (Supplemental Data S1). All cell lines were stored in Tianjin Medical University Cancer Institute and maintained in RPMI-1640 (GIBCO, USA) supplemented with 10% (v/v) fetal bovine serum (FBS) (Gibco, Waltham, MA, USA). Cells were incubated at 37 °C in a humidified atmosphere with 5% CO_2_ (5 L CO_2_/95 L atmospheres). The transfection of *miR-125b* mimic with miRNA scrambled normal control (*mimic-control*) (Shanghai GenePharma, China) was performed according to the manufacturer’s instruction using Lipofectamine RNAi MAX (Invitrogen, CA, USA). The final concentration of miRNA was 50 nM, respectively.

For in vitro experiments, cells were seeded at 3 × 10^5^ per well in the 6-well plates, 5 × 10^4^ per well in the 24-well plates, and 2 × 10^3^ per well in the 96-well plates, and allowed to attach for at least 24 h. To assess the effects of *miR-125b* on cell activities, 50 nM *miR-125b* mimic or scrambled *miRNA* control (*mimic control*) (Shanghai GenePharma, China) were transfected to the cells using the Lipofectamine RNAiMAX (Invitrogen, USA) according to the manufacturer’s instruction. At 6 h post-transfection, culture media were replaced with those containing 10% FBS. *BIK, CASP6*, and *BAK* 3′-UTR were cloned into the pMIR-REPORT Luciferase plasmids (Promega, USA), which were transfected into the GC cells using the DharmaFECT Transfection Reagent (Thermo Scientific, CA, USA). For the functional study of bicalutamide and DHT in vitro, the GC cells were maintained in phenol red-free RPMI 1640 medium (Life Technologies, Carlsbad, CA) with 10% charcoal-dextran-stripped FBS (Bioind, Jerusalem, Israel) for more than 96 h to deplete androgen. Then the cells were treated with DHT (10–100 nM) (Wuhan Dahua Pharmaceutical Co., Ltd, China) and/or 40 mmol/L bicalutamide (Selleck Chemicals, Houston, TX, USA) for the indicated time.

For the functional study of bicalutamide and DHT in vitro, the GC cells were maintained in phenol red-free RPMI 1640 medium (Life Technologies, Inc., Carlsbad, CA) with 10% charcoal-dextran-stripped FBS (Bioind, Jerusalem, Israel) for more than 96 h to deplete androgen. Then the cells were treated with DHT (10–100 nM) (Wuhan Dahua Pharmaceutical Co., Ltd, China) and/or 40 mmol/L bicalutamide (Selleck Chemicals, Houston, TX, USA) for the indicated time.

### Gene expression array for miRNA target genes screening

Gene expression profiling was examined by the Agilent platform (AgilentG3_GX_1color) and analyzed with One-Color Microarray-Based Gene Expression Analysis protocol (Agilent Technologies, USA).

In detail, at 48 h post-transfection of *miR-125b* mimic (50 nM) and mimic control, total RNAs were isolated from cells by Trizol reagents (Invitrogen, USA). The integrity of total RNAs was evaluated by an Agilent 2100 Bioanalyzer (Agilent Technologies, USA). Cy3-labeled target cRNA was prepared by Low Input Quick Amp Labeling Kit (Agilent Technologies, USA) according to the manufacturer’s instructions. Labeled cRNAs were hybridized with a SurePrint G3 Human GE 8 × 60 K Microarrays (Agilent Technologies, USA). Two separate hybridizations were performed for each sample. Array images were captured using a DNA Microarray Scanner (Agilent Technologies, USA), and data were analyzed using Feature Extraction Software (Agilent Technologies, USA) to obtain background-corrected signal intensities.

Data were further analyzed with GeneSpring GX software (Version 11.0, Agilent Technologies) and R package. After data filtering, mRNAs differentially expressed in target cells versus controls were assessed by Fisher’s exact test. Then the Benjamini and Hochberg false discovery rate (FDR) method was used for multiple corrections. Gene sets with an FDR *q* value < 0.05 were considered to be significant. The microRNA target prediction algorithm TargetScan 5.1, was employed for all analyses. Ingenuity pathway analysis (Ingenuity Systems, Redwood City, CA) was employed to assign the biological function to putative target genes of microRNAs with significantly altered expression, utilizing candidate microRNA/mRNA pairs for which an inverse correlation of expression was observed. The output was focused upon canonical pathway gene sets and ranked by statistical significance.

All gene expression data can be found in the Gene Expression Omnibus database under accession number (GSE145959).

### RNA extraction and quantitative real-time PCR

Total RNA was isolated from fresh frozen tissue samples and GC cells by Trizol reagents (Invitrogen, USA). Reverse transcription was performed using TaqMan MicroRNA Reverse Transcription Kit (Applied Biosystems, USA) for microRNA (*miR-125b*) and M-MLV Reverse Transcriptase (Applied Biosystems, USA) for mRNA (BIK, CASP6, and AR) expression level. Small RNA RNU6B and GADPH were utilized as an endogenous control to normalize the level of miRNA and mRNA expression, respectively. The Quantitative real-time PCR probe, primer sequences, and detailed protocol were described in our previous study. The qRT-PCR experiment was conducted using ABI 7900 Real-time PCR (Applied Biosystems, USA) or WaferGen Smartchip platform (WaferGen Biosystems, Inc., USA). Each PCR reaction was performed in triplicate. The average expression levels of microRNA and mRNA in tissues and cells were normalized with the RNU6B and GADPH, respectively, and were calculated using the 2^−△△Ct^ method. Data were analyzed with SDS 2.4 Software (Applied BioSystems, USA). In expression and survival analysis, all gene expression was usually categorized into high- and low-groups using the upper quartile for *miR-125b*, the lower tertile for *CASP6*, the upper tertile for *BIK*, and the median of *AR* as a cutoff.

### Western blot and antibodies

Expression levels of AR, PARP, cleaved PARP, BAX, BCL-2, caspase-6, Flag (DDDDK-tag), BIK, Ki-67, and β-actin proteins in GC cell lines or GC tissues were detected by Western blot analysis according to the protocol of our previous studies. Protein was extracted from tissues and cells at 24 or 48 h post-transfection using RIPA buffer. The protein concentration was measured by using a BCA protein assay kit (Thermo Scientific, CA, USA). Approximately 40 μg of lysates per sample were analyzed using standard western blotting assay procedure. Briefly, the lysates were separated by SDS–PAGE using 10% polyacrylamide gels and transferred onto a PVDF membrane for 1.5 h. The membrane was blocked with 5% skim milk followed by incubation with the primary antibodies summarized in Supplementary Table S7 at 4 °C overnight. After 3 × 10 min washes with TBST at room temperature, the membrane was incubated with HRP-conjugated secondary antibody Mouse Anti-Human (1:2000) (Santa Cruz, USA) for 1 h at room temperature. The detected proteins were visualized using the Visualizer Western Blot Detection Kit (Millipore, USA). Detection was performed by C-DiGit Chemiluminescent Western Blot Scanner (LI-COR, USA).

### MTT assay

Cell proliferation was measured by the MTT assay (Nanjing KeyGEN Biotech, China). After being seeded in the 96-well plates for 24 h, cells were transfected with *miR-125b* mimics or miRNA controls. At 24, 48, 72, and 96 h of post-transfection, cells were gently washed with PBS, and 20 µl MTT (5 mg/ml) was added to the cell culture. After 4 h of incubation, the media were discarded, and 150 µl DMSO was added to each well to dissolve the precipitates. The absorbance of the resulting solution was measured at 590 nm wavelength with a microplate reader (BioTeck, VT, USA). Each experimental condition was carried out in six replicates and repeated three times.

### TUNEL assay of xenograft tumors

Paraffin-embedded samples were analyzed for DNA fragmentation using a TUNEL assay with the Click-iT^™^ TUNEL Colorimetric IHC Detection Kit (Thermo Scientific, CA, USA) according to the manufacturer’s instruction. Briefly, the equilibration buffer was added to slides and incubated for 10 min, followed by 10 min incubation in 20 µg/ml proteinase K solution. The sections were washed in PBS and incubated with TdT enzyme at 37 °C for 1 h in a humidified chamber to incorporate biotinylated nucleotides at the 3′- OH ends of DNA. The slides were incubated in horseradish peroxidase-labeled streptavidin to bind the biotinylated nucleotides, followed by detection with stable chromagen DAB. The images on the slides were visualized, and the apoptotic cells were identified by dark brown cytoplasmic staining.

### Immunofluorescence staining

Cells were seeded onto uncoated glass slide coverslips and cultured in a complete medium under standard cell culture conditions. Cells were fixed in 4% paraformaldehyde at room temperature for 15 min, followed by permeabilization in 1×PBS containing 0.05% Triton-100 for 10 min at room temperature. The cells were blocked in a blocking solution (1×PBS containing 10% normal goat serum) for at least 1 h. After being briefly washed with 1×PBS, the cells were incubated with a mouse monoclonal anti-human E-cadherin antibody (BD, USA) with a dilution of 1:100 in the blocking solution at 4 °C overnight. The cells were washed and then incubated with a goat anti-mouse IgG conjugated with Alexa Fluor 488 (Invitrogen, USA) with a dilution of 1:1000 in the blocking solution at ambient temperature for 1 h. Phalloidin (F-actin) staining was performed at ambient temperature for 45 min.

### Tissue microarray and immunohistochemical staining

BIK, Caspase-6, and AR protein expression in tumor samples were measured with IHC staining. Tissue microarrays (TMA) were constructed from the archived formalin-fixed paraffin-embedded tissue blocks using the “TMA Builder” (Beecher Instruments, USA). A total of 15 slides were constructed, which contained both tumor and adjacent non-tumor tissues from patients with GC. The IHC staining of the TMA slides was performed as previously described^29^.

### Electromobility shift assays (EMSA)

The putative AR binding sites in the *miR-125b* promoter were identified using PROMO3.0 software (http://alggen.lsi.upc.es/cgi-bin/promo_v3/promo/promoinit.cgi?dirDB=TF_8.3). Nuclear proteins from HGC-27 were extracted using NE-PER^™^ Nuclear and Cytoplasmic Extraction Reagents (Thermo Scientific, CA, USA) according to the manufacturer’s instructions. Double-stranded oligonucleotides (Supplemental Table S8) corresponding to the potential AR binding sites were end-labeled with biotin in 5′ terminus. Binding assays were performed in 20 μl of reaction mixture with 5 μg of nuclear protein extracts and 1 nM labeled probes at room temperature for 1 h in binding buffer (10 mM Tris-Cl, 55 mM KCl, 2.5 mM MgCl_2_, 0.25 mM EDTA, 1 mM DTT, 0.05% NP-40, 5% Glycerol and 1 μg poly dI-dC). Reactions were separated on 10% Tris-Borate-EDTA (TBE) polyacrylamide gels (Bio-Rad) in TBE buffer at 100 V for 40 min. Duplex-bound complexes were transferred onto Zeta-Probe positively-charged nylon membranes (Bio-Rad, USA) by semi-dry transfer at 10 mA for 30 min, then crosslinked onto the membranes under 254 nm ultra-violet light for 15 min. After crosslinked, Membranes were processed with the LightShift Chemiluminescent EMSA kit (Thermo Scientific, CA, USA) as per the manufacturer’s instructions, and chemiluminescent signals were visualized with GBOX/Chemi XT4 Gel Documentation System (Syngene, Cambridge, UK).

### Lentivirus construction and infection

Lentiviral particles expressing AR full-length cDNA sequence (AR-FLAG in GV358 vector) were constructed by Genechem (Shanghai Genechem Co., LTD). MGC-803 cells were infected by incubation with viral supernatants for 12 h at 37 °C. Cells were subsequently placed under puromycin (5 μg/mL) for the selection of stable overexpressing AR cells for 2 weeks. AR expression (GenBank: NM_000044) was assessed by qRT-PCR and western blot analysis. Corresponding empty GV358 vector was used as a negative control.

### Animals, orthotopic in vivo model, and tissue processing

All xenograft experiments were approved and supervised by the Tianjin Cancer Hospital Institutional Animal Care and Use Committee. The BALB/c nude mice were provided by the Model Animal Research Center of Nanjing University and maintained in the Animal Center of Tianjin Cancer Hospital Institute under specific pathogen-free conditions and bred in-house. The mice were cared for according to Chinese animal welfare legislation and under the NIH Guidelines of Care and Use of Laboratory Animals.

For the **xenografted tumor model of drug effect study**, 2 × 10^6^ cells from each gastric cell line (HGC-27 and MGC-803) were re-suspended in 200 μl medium and subcutaneously injected into the nude mice. Above 3 weeks after tumor cell inoculation, when the tumors reached a volume of ~150–200 mm^3^, the tumor tissues were harvested and macro dissected to minimize the content of necrotic tissue. Then the tumor pieces were immediately placed in 10 ml of Dulbecco’s Phosphate Buffered Saline (DPBS) with 20 mg/ml of Gentamicin for 5 min, then rinsed with DPBS and cut into 20 mg pieces (about 3–4 mm per cubed side) for implantation. Tumor bits were implanted subcutaneously in 6–8 weeks old female and male athymic Nu/Nu mice. Treatment was initiated at 14 days post-tumor inoculation when the solid tumors reached 80–100 mm^3^ in size. Both male and female nude mice were randomly assigned to the control or experimental group and treated by oral gavage with either vehicle alone (control group) or bicalutamide (experimental group) in sesame oil at 50 mg/kg once daily (Q.D.). All female mice were received concomitant DHT administration (10 mg/Kg) by oral gavage (Q.D.). The volume of subcutaneous tumors was measured every 3 or 4 days by external caliper and was calculated using the ellipsoid formula: volume = 1/2 (Length × Width^2^). The mice were euthanized by carbon dioxide asphyxiation and cervical dislocation. The tumors were harvested, and the weight, number, and location of the tumor were recorded. The body weights were similar among groups, demonstrating that drinking and feeding habits were not influenced. Tumor tissue was snap-frozen or fixed in formalin. H&E, IHC staining, and TUNEL staining were performed on formalin-fixed, paraffin-embedded slides.

For the **miRNA transfection model**, we performed the experiment according to our previously established method^29^. In brief, all animals were 8–12 weeks of age at the time of injection. MGC-803 and SGC-7901 cells were injected subcutaneously on the mouse back (5 × 10^6^ cells/animal). Seven days after tumor cell injection, mice were randomly separated into two groups and injected with microRNAs (miRNA control or *miR-125b*) incorporated with vivo-jetPEI^®^ (Polyplus-transfection, Illkirch, France) according to the manufacturer’s instructions. Twice-weekly treatments with microRNAs lasted for 4–6 weeks. We measured the volume of subcutaneous tumors in vivo by external caliper twice every week. The tumor volume was calculated using the modified ellipsoid formula 1/2 (Length × Width^2^). Volumes of a group of tumors were determined independently by two observers to assess inter-observer variation. All mice in the experiment were then killed and necropsied, and their tumors were harvested. The weight, number, and location of the tumor were recorded. The body weights were similar between groups, suggesting that feeding and drinking habits were not affected. Tumor tissue was snap-frozen or fixed in formalin. H&E and IHC staining were performed on formalin-fixed, paraffin-embedded slides.

### PDX model

The PDX models were established by LIDE Biotech (Shanghai, China) as described previously^59^. All study protocols were reviewed and approved by the Institutional Animal Care and Use Committee (IACUC) and Institutional Ethics Committee at Shanghai LIDE Biotech. All PDX animal experiments complied with established national and international ethical regulations for laboratory animal protection. Tumor tissue acquisition was approved by the ethics committees of each participating hospital and agreed upon by each patient via written informed consent. All the procedures related to these GC samples were carried out according to ethical regulations on the experimental use of human tissues.

GC samples were obtained from two female patients (PDX-494 and PDX-132) who received gastric adenocarcinoma diagnosis. The AR expression were detected by IHC staining on P0 or P1 generation of PDX tumor. We used CB17-SCID mice for PDX model recovery and nu/nu mice (Beijing Vitonlihua Experimental Animal Technology Co. Ltd, Beijing, China) for anti-AR drug efficacy tests. For PDX xenografting, in vivo drug treatment, histologic and immunofluorescence studies, all manipulations were performed and followed by the protocol of cell lines derived xenograft described above.

Briefly, immune-deficient nu/nu male mice were inoculated in the right flank with P1-P3 PDX tumor fragments. When the tumors reached 100–200 mm^3^, the mice were randomly segregated into control, bicalutamide (BIC, APExBIO Technology) and S-1 (Tegafur, Gimeracil and Oteracil Potassium Capsules from Jiangsu Hengrui Medicine Co., Ltd.) group, with 5 or 6 mice with similar average tumor volume. Bicalutamide was administered via oral gavage at a dose of 50 mg/kg/mouse per day for 4 weeks. The chemotherapeutic agent S-1 (a 5-fluorouracil (5-FU) analog) group was set as a positive control of drug efficiency for PDX-494 model. S-1 was administered orally five times weekly for 3 weeks at doses of 10 mg/kg. Vehicle (4% absolute ethanol, 5% Tween-80, 5% propylene glycol in 86% water) was administered orally to the control group one time per day (Q.D.) for 4 weeks. The expression of AR and ki-67 were detected by IHC staining in paraffin-embedded tumor tissue sections. Apoptosis analysis was performed using a fluorescence TUNEL apoptosis detection kit (Millipore) according to the manufacturers instructions.

### Statistical analysis

All experiments were performed in triplicate. All statistical analyses were carried out with SPSS20.0 for IBM (SPSS Inc) and GraphPad Prism 7.0 statistic software. The expression levels of *miR-125b* and *AR* were log2^−△△ct^ transformed and analyzed as a continuous variable by means and standard deviations (mean ± SD). *X*^2^ test was used to compare the differences in gene expression by clinicopathological features of patients. A paired *t*-test was used to analyze the differences in *miR-125b* or *AR* expression between GC and paired noncancerous tissues. The correlation between the expression of *miR-125b* and *AR* in tissue from GC patients used the Pearson correlation test. The LASSO Cox regression model was used to select the most significant prognostic AR-related markers of GC and analyzed using the glmnet package^60^. The Kaplan–Meier method was used for survival analysis, and the differences in survival were measured using the log-rank test. Associations between the expression of *miR-125b*, *AR*, *BIK*, and *CASP6* and GC survival were also evaluated with the Cox proportional hazards regression model at both univariate and multivariate levels. The in vitro experiments were analyzed by independent sample *t*-test or one-way ANOVA. Differences were considered statistically significant when a *p* value was <0.05, and all *P* values reported were two-sided.

## Conclusion

In conclusion, AR/miR-125b is activated by androgen, elevated levels of miR-125b circuit repress the apoptosis pathway by targeting pro-apoptosis genes in GC. The AR antagonist, bicalutamide, attenuated the AR/miR-125b axis, induced pro-apoptosis genes, and lead to cancer cell apoptosis and growth inhibition.

## Supplementary information

Supplementary Materials

Supplementary Table S2

Supplementary Table S4

Supplementary Table S5

## Data Availability

The datasets generated and analyzed during the current study are available in Gene Expression Omnibus (accession number GSE145959). Other datasets used and/or analyzed during the current study are available from the corresponding author on reasonable request.

## References

[1] Bray F (2018). Global cancer statistics 2018: GLOBOCAN estimates of incidence and mortality worldwide for 36 cancers in 185 countries. CA Cancer J. Clin..

[2] Chandanos E, Lagergren J (2008). Oestrogen and the enigmatic male predominance of gastric cancer. Eur. J. Cancer.

[3] Cancer Genome Atlas Research N. (2014). Comprehensive molecular characterization of gastric adenocarcinoma. Nature.

[4] Cristescu R (2015). Molecular analysis of gastric cancer identifies subtypes associated with distinct clinical outcomes. Nat. Med.

[5] Polom K (2018). Meta-analysis of microsatellite instability in relation to clinicopathological characteristics and overall survival in gastric cancer. Br. J. Surg..

[6] Conforti F (2018). Cancer immunotherapy efficacy and patients’ sex: a systematic review and meta-analysis. Lancet Oncol..

[7] Lordick F, Shitara K, Janjigian YY (2017). New agents on the horizon in gastric cancer. Ann. Oncol..

[8] Yuan Y (2016). Comprehensive Characterization of Molecular Differences in Cancer between Male and Female Patients. Cancer Cell.

[9] Fu BC (2020). Height as a mediator of sex differences in cancer risk. Ann. Oncol..

[10] Qiu MZ (2011). Clinicopathological characteristics and prognostic analysis of gastric cancer in the young adult in China. Tumour Biol..

[11] Sipponen P, Correa P (2002). Delayed rise in incidence of gastric cancer in females results in unique sex ratio (M/F) pattern: etiologic hypothesis. Gastric Cancer.

[12] Jeong O, Park YK (2011). Clinicopathological features and surgical treatment of gastric cancer in South Korea: the results of 2009 nationwide survey on surgically treated gastric cancer patients. J. Gastric Cancer.

[13] Huh CW (2013). Signet ring cell mixed histology may show more aggressive behavior than other histologies in early gastric cancer. J. Surg. Oncol..

[14] Kim HW (2016). Sex Disparity in Gastric Cancer: female Sex is a Poor Prognostic Factor for Advanced Gastric Cancer. Ann. Surg. Oncol..

[15] Kitamura K, Taniguchi H, Yamaguchi T, Takahashi T (1996). Early gastric cancer in young adults. Tohoku J. Exp. Med.

[16] Theuer CP (1996). Gastric adenocarcinoma in patients 40 years of age or younger. Am. J. Surg..

[17] Ahmed SMH (2020). Fitness trade-offs incurred by ovary-to-gut steroid signalling in Drosophila. Nature.

[18] Wang Q, Carroll JS, Brown M (2005). Spatial and temporal recruitment of androgen receptor and its coactivators involves chromosomal looping and polymerase tracking. Mol. Cell.

[19] Yamane K (2006). JHDM2A, a JmjC-containing H3K9 demethylase, facilitates transcription activation by androgen receptor. Cell.

[20] Metzger E (2005). LSD1 demethylates repressive histone marks to promote androgen-receptor-dependent transcription. Nature.

[21] Mc Menamin UC (2018). Hormonal and reproductive factors and risk of upper gastrointestinal cancers in men: a prospective cohort study within the UK Biobank. Int J. Cancer.

[22] Xia N, Cui J, Zhu M, Xing R, Lu Y (2019). Androgen receptor variant 12 promotes migration and invasion by regulating MYLK in gastric cancer. J. Pathol..

[23] Gan L (2012). Expression profile and prognostic role of sex hormone receptors in gastric cancer. BMC Cancer.

[24] Kim YS, Noh HJ, Yoo I, Lee D (2020). Androgen Receptor is Mostly Not Expressed in Gastric Cancers. Appl. Immunohistochem. Mol. Morphol..

[25] Tian Y (2013). Androgen receptor may be responsible for gender disparity in gastric cancer. Med. Hypotheses.

[26] Wang R (2019). Androgen Receptor Promotes Gastric Carcinogenesis via Upregulating Cell Cycle-Related Kinase Expression. J. Cancer.

[27] Thomas H (2017). Colorectal cancer: miR-100 and miR-125b induce cetuximab resistance in CRC. Nat. Rev. Gastroenterol. Hepatol..

[28] Banzhaf-Strathmann J, Edbauer D (2014). Good guy or bad guy: the opposing roles of microRNA 125b in cancer. Cell Commun. Signal.

[29] Song F (2014). Integrated microRNA network analyses identify a poor-prognosis subtype of gastric cancer characterized by the miR-200 family. Clin. Cancer Res..

[30] Brown CJ (1991). A gene from the region of the human X inactivation centre is expressed exclusively from the inactive X chromosome. Nature.

[31] Dunford A (2017). Tumor-suppressor genes that escape from X-inactivation contribute to cancer sex bias. Nat. Genet..

[32] Fang H, Disteche CM, Berletch JB (2019). X Inactivation and Escape: epigenetic and Structural Features. Front. Cell Dev. Biol..

[33] Haupt S (2019). Identification of cancer sex-disparity in the functional integrity of p53 and its X chromosome network. Nat. Commun..

[34] Chaligne R (2015). The inactive X chromosome is epigenetically unstable and transcriptionally labile in breast cancer. Genome Res..

[35] Pageau GJ, Hall LL, Ganesan S, Livingston DM, Lawrence JB (2007). The disappearing Barr body in breast and ovarian cancers. Nat. Rev. Cancer.

[36] Winham SJ (2019). Molecular signatures of X chromosome inactivation and associations with clinical outcomes in epithelial ovarian cancer. Hum. Mol. Genet.

[37] Schweizer MT, Yu EY (2017). AR-Signaling in Human Malignancies: prostate Cancer and Beyond. Cancers (Basel)..

[38] Chiu CM (2007). Hepatitis B virus X protein enhances androgen receptor-responsive gene expression depending on androgen level. Proc. Natl Acad. Sci. USA.

[39] Wu JT, Han BM, Yu SQ, Wang HP, Xia SJ (2010). Androgen Receptor Is a Potential Therapeutic Target for Bladder Cancer. Urology.

[40] Miyamoto H (2007). Promotion of bladder cancer development and progression by androgen receptor signals. J. Natl Cancer Inst..

[41] Jing JC (2018). KDM4B promotes gastric cancer metastasis by regulating miR-125b-mediated activation of Wnt signaling. J. Cell Biochem..

[42] Li X (2011). miRNA-223 promotes gastric cancer invasion and metastasis by targeting tumor suppressor EPB41L3. Mol. Cancer Res..

[43] Sui M (2017). Upregulation of miR-125b is associated with poor prognosis and trastuzumab resistance in HER2-positive gastric cancer. Exp. Ther. Med.

[44] Ottaviani S (2018). TGF-beta induces miR-100 and miR-125b but blocks let-7a through LIN28B controlling PDAC progression. Nat. Commun..

[45] Sun D (2014). Regulation of several androgen-induced genes through the repression of the miR-99a/let-7c/miR-125b-2 miRNA cluster in prostate cancer cells. Oncogene.

[46] Lu Y (2017). lncRNA MIR100HG-derived miR-100 and miR-125b mediate cetuximab resistance via Wnt/beta-catenin signaling. Nat. Med..

[47] Zhou JN (2015). MicroRNA-125b attenuates epithelial-mesenchymal transitions and targets stem-like liver cancer cells through small mothers against decapentaplegic 2 and 4. Hepatology.

[48] Wei X (2021). MiR-125b Loss Activated HIF1alpha/pAKT Loop, Leading to Trans-Arterial Chemoembolization Resistance in Hepatocellular Carcinoma. Hepatology.

[49] Dong H (2019). Activation of LncRNA TINCR by H3K27 acetylation promotes Trastuzumab resistance and epithelial-mesenchymal transition by targeting MicroRNA-125b in breast Cancer. Mol. Cancer.

[50] Bianchini G, Balko JM, Mayer IA, Sanders ME, Gianni L (2016). Triple-negative breast cancer: challenges and opportunities of a heterogeneous disease. Nat. Rev. Clin. Oncol..

[51] Kanda T (2017). Androgen Receptor Could Be a Potential Therapeutic Target in Patients with Advanced Hepatocellular Carcinoma. Cancers.

[52] Kono M (2017). Androgen Receptor Function and Androgen Receptor-Targeted Therapies in Breast Cancer: a Review. JAMA Oncol..

[53] Elattar A (2012). Androgen receptor expression is a biological marker for androgen sensitivity in high grade serous epithelial ovarian cancer. Gynecol. Oncol..

[54] Huang G., Liu z., Maaten Lvd., Weinberger KQ. Fast gene set enrichment analysis. *arXiv* 2016: arXiv:1608.06993 [cs.CV].

[55] Sundararajan M., Taly A., Yan Q. Axiomatic Attribution for Deep Networks. *arXiv* 2017: preprint arXiv:1703.01365.

[56] Robinson MD, McCarthy DJ, Smyth GK (2010). edgeR: a Bioconductor package for differential expression analysis of digital gene expression data. Bioinformatics.

[57] Chen C., Chen H., He Y., Xia R. TBtools - an integrative toolkit developed for interactive analyses of big biological data. *bioRxiv* 2020, bioRxiv [Preprint] 10.1101/289660. 10.1016/j.molp.2020.06.009 32585190

[58] Zhou Y (2019). Metascape provides a biologist-oriented resource for the analysis of systems-level datasets. Nat. Commun..

[59] Zhang F (2018). Characterization of drug responses of mini patient-derived xenografts in mice for predicting cancer patient clinical therapeutic response. Cancer Commun. (Lond.).

[60] Zhang JX (2013). Prognostic and predictive value of a microRNA signature in stage II colon cancer: a microRNA expression analysis. Lancet Oncol..

