# Supplementary material for: Interrogation of gender disparity uncovers androgen receptor as the transcriptional activator for oncogenic miR-125b in gastric cancer
Source: Cell Death Dis. 2021 May 4;12(5):441. doi: 10.1038/s41419-021-03727-3 (PMC8096848; doi:10.1038/s41419-021-03727-3)
Supplement: Supplementary file 1 — Supplementary Materials [file 41419_2021_3727_MOESM1_ESM.docx]

**Supplementary Materials for**

**Interrogation of gender disparity uncovers androgen receptor as the transcriptional activator for oncogenic *miR-125b* in gastric cancer**

Ben Liu, Meng Zhou, Xiangchun Li, Xining Zhang, Qinghua Wang, Luyang Liu, Meng Yang, Da Yang, Yan Guo, Qiang Zhang, Hong Zheng, Qiong Wang, Lian Li, Xinlei Chu, Wei Wang, Haixin Li, Fengju Song, Yuan Pan, Wei Zhang and Kexin Chen

Correspondence to: [chenkexin@tmu.edu.cn](mailto:chenkexin@tmu.edu.cn)

**This PDF file includes:**

**Supplemental Figures. S1 to S9**

Fig. S1. Relates to Figure 1

Fig. S2. Relates to Figure 2

Fig. S3. Relates to Figure 2

Fig. S4. Relates to Figure 3

Fig. S5. Relates to Figure 4

Fig. S6. Relates to Figure 4

Fig. S7. Relates to Figure 5

Fig. S8. Relates to Figure 6

Fig. S9. Relates to Figure 7

**Supplemental Tables S1, S3, S6-S8**

Table S1. Relates to Fig. 1 and Fig. 2

Table S3. Relates to Fig. 1, Fig. 2 and Fig. 4

Table S6. Relates to Fig. 4

Table S7. Relates to Materials and Methods

Table S8. Relates to Materials and Methods

The captions for Table S2, S4, S5

The captions for Data S1 (cell line STR authentication reports)

**Other Supplementary Materials for this manuscript include the following:**

Table S2. Relates to Fig. 1 (Provided as a separated Excel file)

Table S4. Relates to Fig. 4 (Provided as a separated Excel file)

Table S5. Relates to Fig. 4 (Provided as a separated Excel file)

**Supplemental Figures**

**Fig. S1**

**
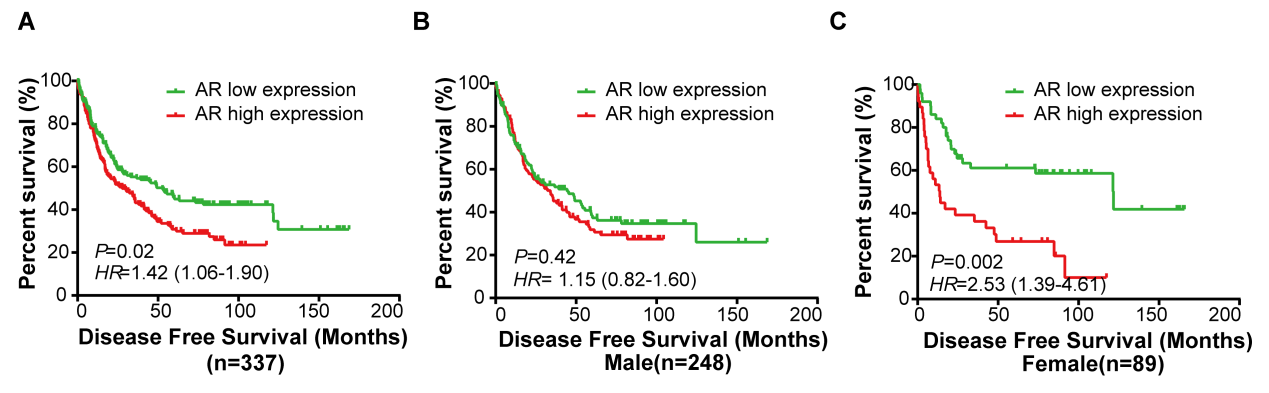
**

**Supplemental Figure S1. (Related to Figure 1) Association of expression of *AR* with gastric cancer disease-free survival (DFS).** (A). Kaplan-Meier curves for DFS of 337 GC patients. (B and C) Kaplan-Meier analysis of DFS according to low and high AR expression in 248 male cases (B) and 89 female cases (C).

**Fig. S2**

**
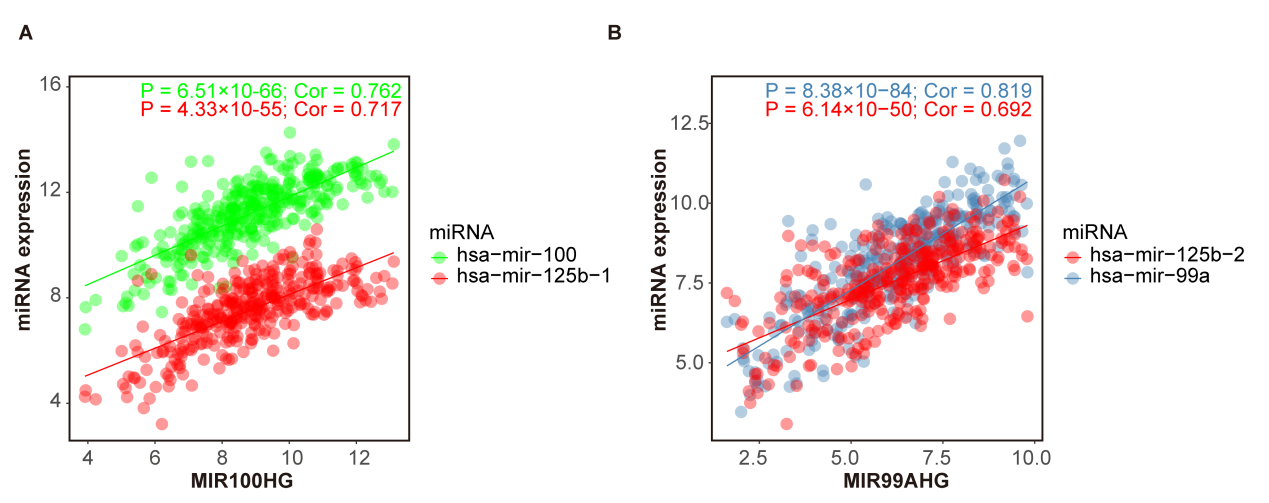
**

**Supplemental Figure S2. (Related to Figure 2)** Scatter plots depict the correlation of AR-related lncRNA cluster and miRNA from TCGA GC dataset analysis. (A) Correlation of MIR100HG versus miR-125b-1 or miR-100. (B) Correlation of MIR99AHG versus miR-125b-2 or miR-99a (right panel) in Tianjin dataset. *r*：Pearson correlation coefficients.

**Fig. S3**


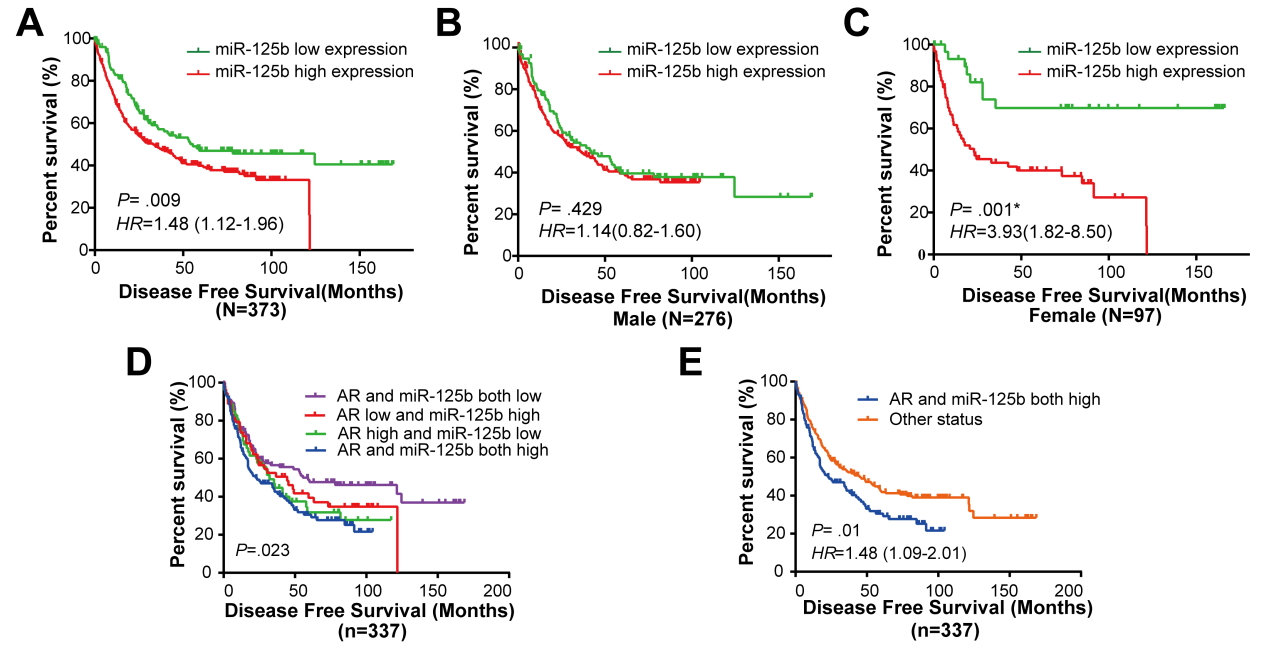


**Supplemental Figure S3. (Related to Figure 2) Association of expression of *AR* and *miR-125b* with gastric cancer disease-free survival (DFS).** (A). Kaplan-Meier curves for DFS of 373 GC patients. (B and C) Kaplan-Meier analysis of DFS according to low and high miR-125b expression in 276 male cases (B) and 97 female cases (C). (D) Kaplan-Meier analysis of DFS in four groups according to low and high AR and *miR-125b* expression. (E) Kaplan-Meier analysis of DFS in two groups according to low and high AR and *miR-125b* expression. In addition to the group of AR/*miR-125b* both high expression, the other three groups in panel D were merged into one group.

**Fig. S4**

**
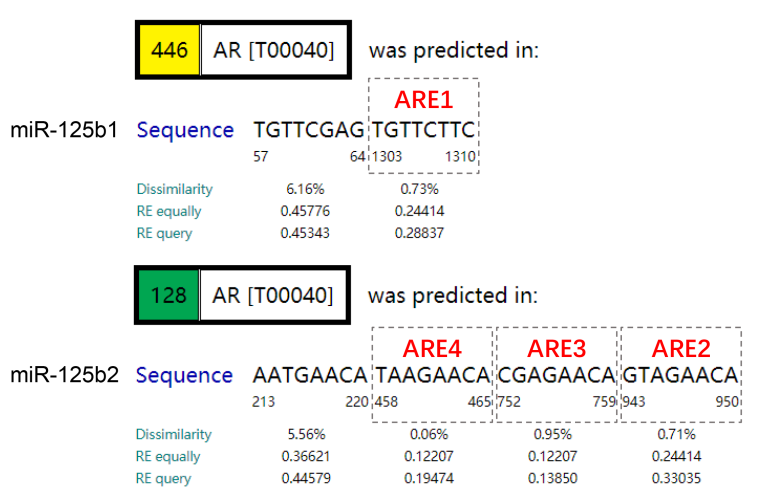
**

**Supplemental Figure S4. (Related to Figure 3) The predicted AREs on the promoter of *miR-125b*.** Predicted result showing that the 5′ UTRs (2kb upstream of the transcriptional start site)of the human *miR-125b*-1 and *miR-125b*-2 promoter region contain one (ARE1, -1303/-1310) and three (ARE2, -943/-950; ARE3, -752/-759; ARE4, -458/-465) AREs, respectively. <http://alggen.lsi.upc.es/cgi-bin/promo_v3/promo/promoinit.cgi?dirDB=TF_8.3>

**Fig. S5**

**
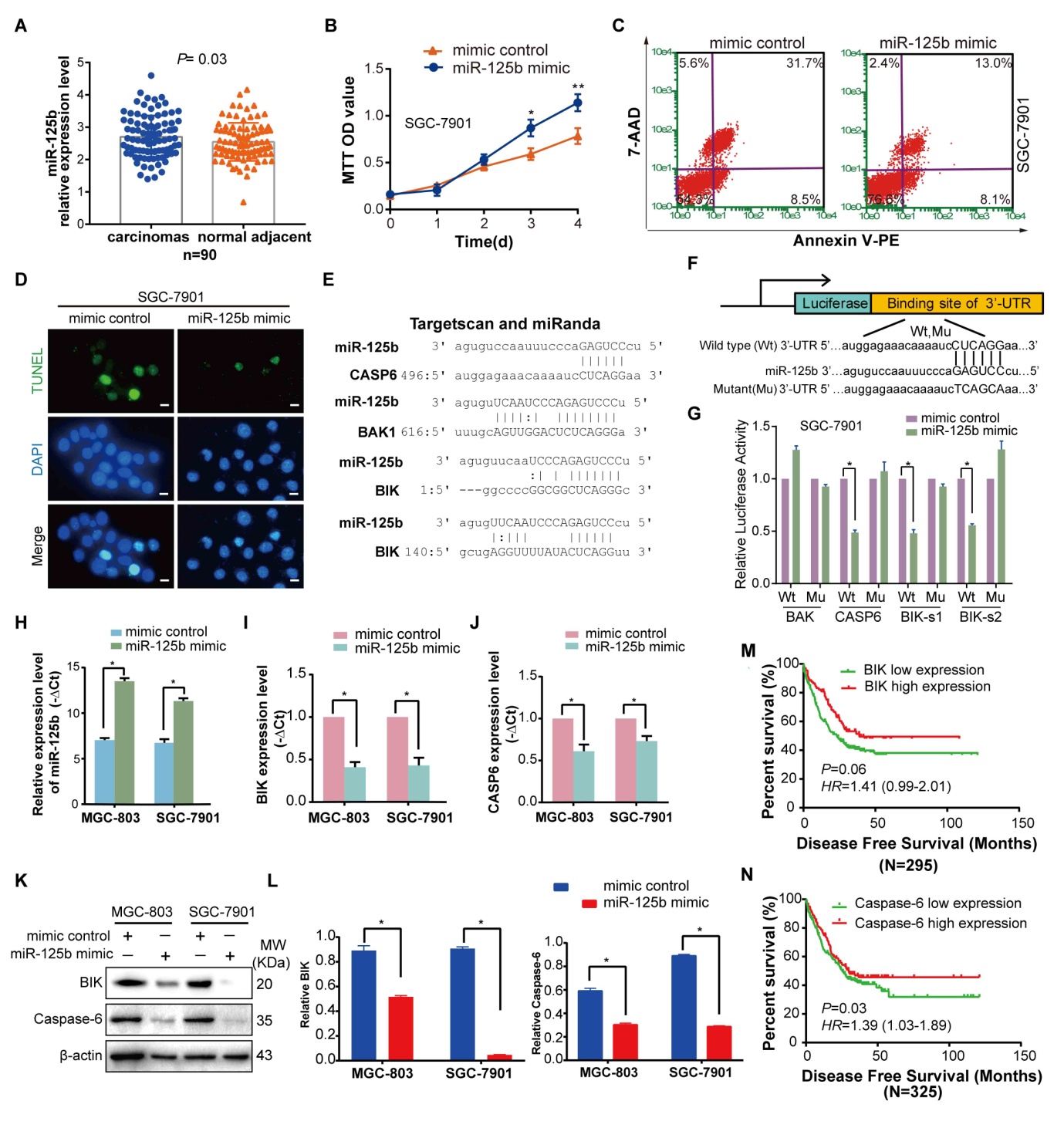
**

**Supplemental Figure S5. (Related to Figure 4) *MiR-125b* suppressed cell apoptosis and promoted proliferation in gastric cancer.** (A) Differential expression of the miR-125b gene in gastric cancer and adjacent normal tissues. miR-125b expression levels in 90 pairs of tumor and paired adjacent normal tissues measured by real-time RT-PCR (TaqMan) and RNU6B was used as an internal control. (B) SGC-7901 cell lines were transfected with *miR-125b* mimic or mimic control, and the MTT assay measured cell proliferation. (C) Representative dot plots of Annexin V/7-AAD staining. After transfected with *miR-125b* mimic or mimic control for 48 h, the GC cells were incubated for 24 h with 40 μM etoposide. Then GC cells were stained with AnnexinV/7-AAD dual staining solutions and detected by flow cytometry (FCM). Values in the images indicate the percentage of each fraction. (D) Identification of late apoptosis by fluorescent TUNEL assay after transfection of *miR-125b* and mimic control for 48 h and incubated with 40μM etoposide for 24 h in SGC-7901 cells. The stained cells were observed under a fluorescence microscope. The Positive cells show a bright green nucleus and DAPI staining was used to show the location of the nucleus. Columns in panel D represent the TUNEL positive cell number in *miR-125b* and control group. Scale bar=20 µm (E) The predicted binding sites of *miR-125b* in the representative apoptosis genes. (F) Schematic presentation of the reporter plasmid used to illustrate the effect of the *miR-125b* to the 3’-UTR of candidate target genes on luciferase activity. A putative *miR-125b*-binding site exists in the 3’-UTR of the *CASP6* mRNA (wild type, Wt), and 6-nucleotide mutation (Mutant, Mu) were generated in the binding site. (G) Effect of *miR-125b* over-expression on a dual-luciferase reporter plasmid containing the 3′ UTR of candidate target genes (BAK, CASP6, and BIK) was analyzed. The GC cells were co-transfected with either the wild type pMIR-Wt-3’-UTR- BAK/CASP6/BIK (Wt) or corresponding mutant 3’UTR (Mu) or an empty vector and *miR-125b* or mimic-control. Firefly and renilla luciferases were measured in SGC-7901 cell lysate. (H) *Mimic control* was used as a negative control in all the experiments. The impact of *miR-125b* on targeted gene expression was normalized and compared to those of negative miRNA (n=3, *P*< 0.001). (I and J) Three GC cells were transfected with the *miR-125b* mimic or mimic-control. qRT-PCR analysis for BIK (I) and CASP6 (J) expression was performed. GAPDH was used as an internal control. (K-L) The protein level of BIK and Caspase-6 was decreased in two GC cell lines when transfected with *miR-125b* with beta-actin as a loading control. Western blots (K) and quantified results (L) of BIK and Caspase-6 protein expression are demonstrated. * *P*< 0.05 vs. control group. (M-N) Kaplan-Meier disease-free survival curves according to low and high BIK (M) and Caspase-6 (N) protein expression in 295 and 325 cases, respectively. Green and red lines represent low and high protein expression, respectively.

**Fig. S6**


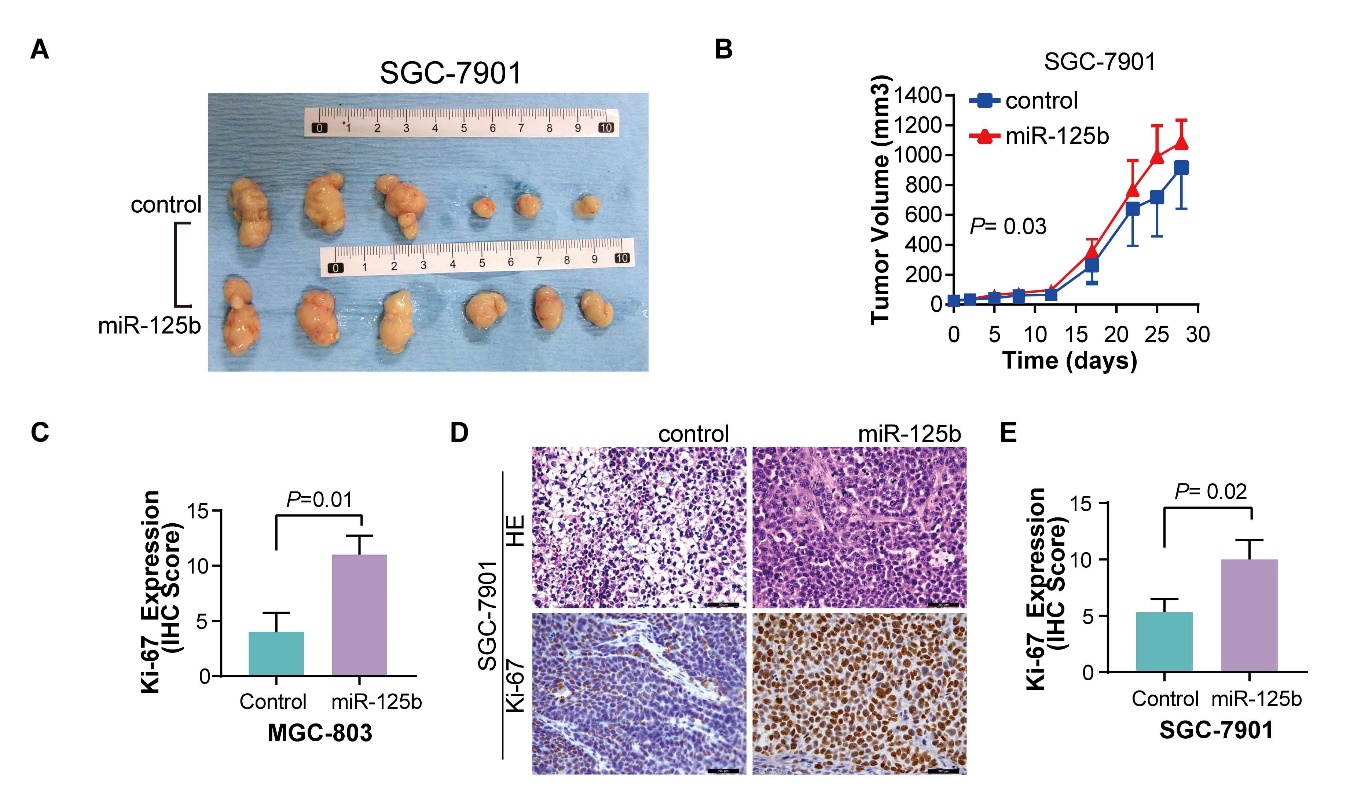


**Supplemental Figure S6.** **(Related to Figure 4) *miR-125b* overexpression promotes tumorigenesis of GC xenografts in vivo.** (A) Representative images of tumor sizes in control and miR-125b treated mice from SGC-7901 cells. (B) In vivo xenograft tumor growth curve of SGC-7901 cells expressing control and miR-125, error bars represent ± SEM. (C) Quantification of Ki-67 protein expression in MGC-803 cellls. Error bars represent ±SD. * indicates significant difference *P*< 0.05 (D) the representative images of HE and Ki-67 staining of SGC-7901 xenograft tumors after 4 weeks of *miR-125b* and control in vivo transfection. Scale bar= 50 µm (E) Quantification of Ki-67 protein expression in SGC-7901 cellls. Error bars represent ±SD. * indicates significant difference *P*< 0.05

**Fig. S7**


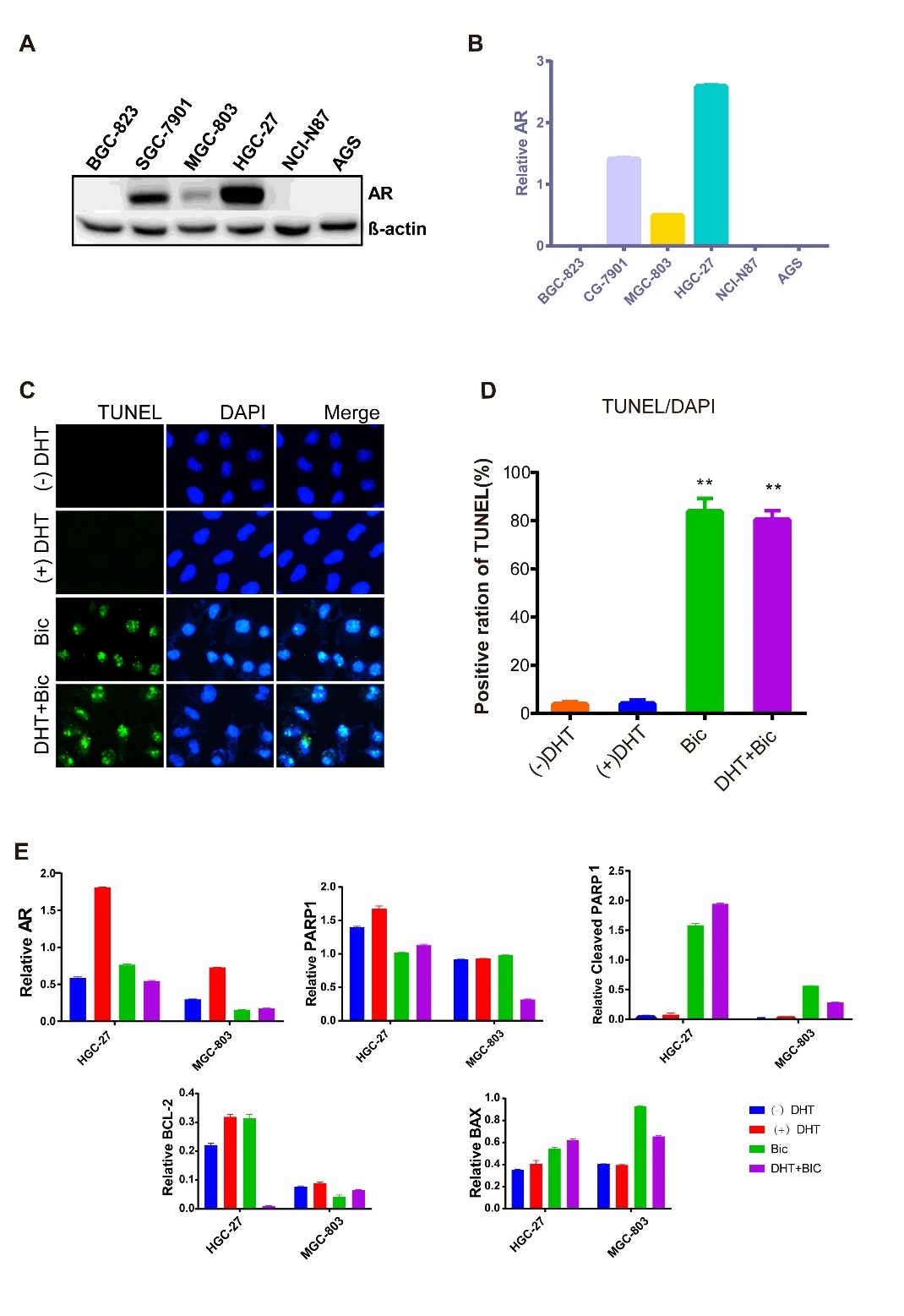


**Supplemental Figure S7. (Related to Figure 5) The expression of AR in gastric cancer cell lines and TUNEL assay for detecting the effect of Bicalutamide on apoptosis of MGC-803 cells.** (A) Western blot assay detected AR expression in BGC-823, SGC-7901, MGC-803, HGC-27, NCI-N87, and AGS GC cells. (B) Quantified results of western blots in panel A. (C)  Identification of late apoptosis by fluorescent TUNEL assay after treatment with Bic and/or DHT for 48 hr in MGC-803 cells. (D) Columns represent the TUNEL positive cell numbers in panel A. magnification, 200×. (E) Quantified results of western blots in Fig. 5J.

**Fig. S8**


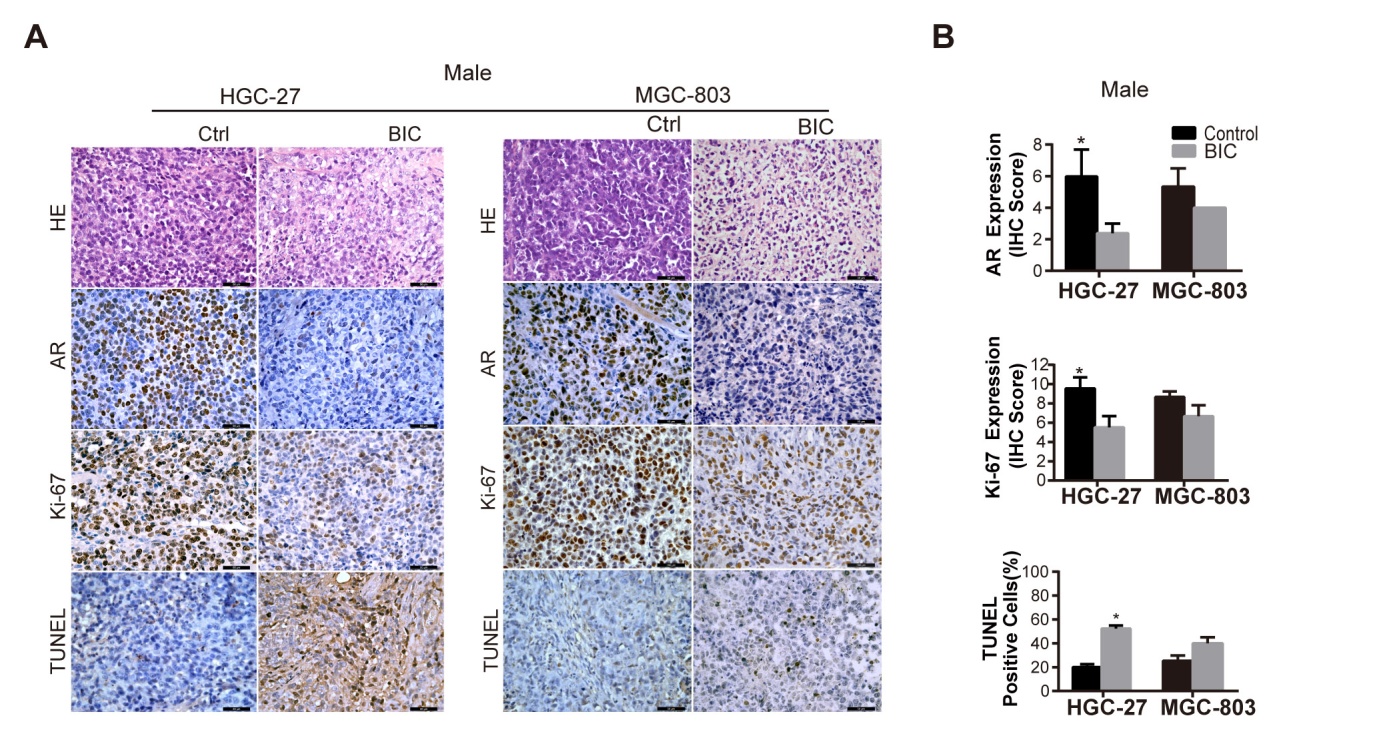


**Supplemental Figure S8. (Related to Fig. 6) Bicalutamide (Bic) decreases cellular viability and increases necrosis and apoptosis in HGC-27 and MGC-803 xenografts.** **(A)** The AR and Ki-67 protein expression and apoptosis status of MGC-803 (left panel) and HGC-27 (right panel) tumor samples from control and BIC treated male mice were evaluated by HE staining, immunohistochemistry (IHC), and TUNEL assay. Scale bars represent 50 μm. **(B)** Quantification of AR and Ki-67 protein expression and TUNEL positive cell in MGC-803 and HGC-27 tumor samples in male mice model. Error bars represent ±SD.

**Fig. S9**


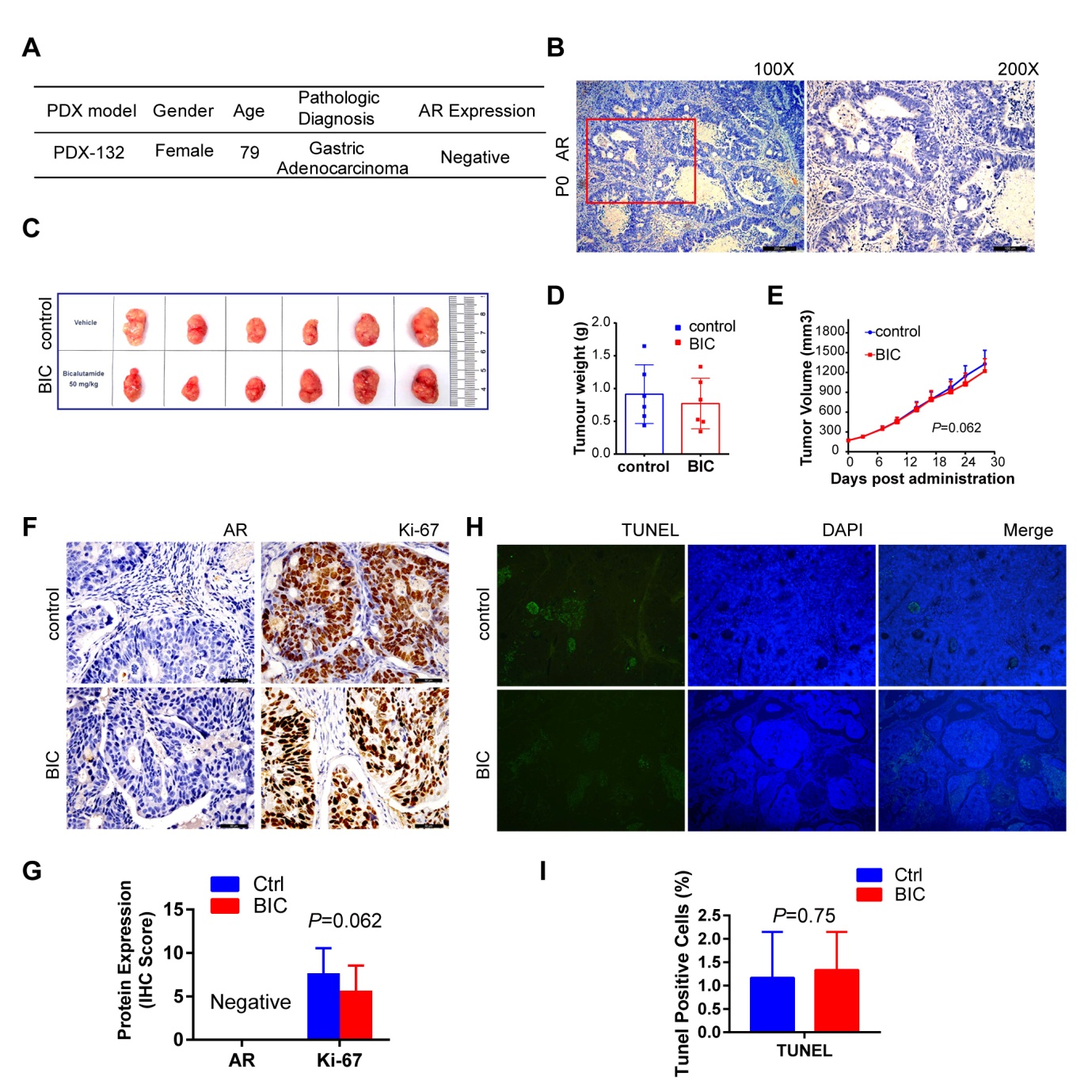


**Supplemental Figure S9. (Related to Fig. 7) In vivo antitumor activity of Bicalutamide (Bic) was detected in PDX-132 mice model from AR negative female GC patient .**

**(A)** Patient Information of PDX-132 are shown in the table. **(B)** AR expression in P1 (the first passage) of PDX-132 was measured by immunohistochemical staining. Scale bars, 200 μm (left panel) and 100 μm (right panel), respectively. **(C)** Representative images of tumor nodules in control and BIC (50mg/Kg/Day) of the PDX model. Scale bar represents 1 cm. **(D)** Quantification of tumor nodule weights in control and BIC administrate group of PDX model. Error bars represent ± SD. **(E)** The growth curve of tumor xenografts of control and BIC administrate group in PDX model, error bars represent ± SEM. **(F)** The AR and Ki-67 protein expression in PDX model, tumor samples from control and BIC treated mice were evaluated by immunohistochemistry (IHC). Scale bars represent 50 μm. **(G)** Quantification of AR and Ki-67 protein expression in PDX-132 model. **(H)** Apoptotic cells were detected using the TUNEL immunofluorescence technique. Scale bars represent 50 μm. **(I) Q**uantification of apoptosis status in PDX-494 model from control and BIC treated mice was evaluated by TUNEL assay.

**Supplemental Tables**

**Table S1 Association of *miR-125b* and AR expression with clinicopathological features of gastric cancer patients**

| Variable | *MiR-125b* expression(n=373) | | | |  | *AR* expression(n=337) | | | |
| --- | --- | --- | --- | --- | --- | --- | --- | --- | --- |
|  | **Total^a^** | **Low** | **High** | ***P*** |  | **Total** | **Low** | **High** | ***P*** |
| Gender | | | | | | | | | |
| Male | 276 | 96 | 180 | 0.892 |  | 248 | 118 | 130 | 0.164 |
| Female | 97 | 33 | 64 |  |  | 89 | 50 | 39 |  |
| Age | | | | | | | | | |
| < 60 | 171 | 50 | 121 | 0.046* |  | 153 | 74 | 79 | 0.619 |
| ≥ 60 | 202 | 79 | 123 |  |  | 184 | 94 | 90 |  |
| Smoke | | | | | | | | | |
| Yes | 179 | 69 | 110 | 0.039* |  | 165 | 90 | 75 | 0.089 |
| No | 190 | 54 | 136 |  |  | 168 | 76 | 92 |  |
| Alcohol drink | | | | | | | | | |
| Yes | 216 | 84 | 132 | 0.030* |  | 199 | 106 | 93 | 0.121 |
| No | 144 | 40 | 104 |  |  | 126 | 56 | 70 |  |
| Path^b^ | | | | | | | | | |
| Path1 | 100 | 46 | 54 | 0.023* |  | 90 | 54 | 36 | 0.109 |
| Path2 | 84 | 21 | 63 |  |  | 76 | 37 | 39 |  |
| Path3 | 175 | 57 | 118 |  |  | 159 | 73 | 86 |  |
| Path4 | 14 | 5 | 9 |  |  | 12 | 4 | 8 |  |
| Differentiation | | | | | | | | | |
| Well/Moderately | 99 | 35 | 64 | 0.871 |  | 92 | 47 | 45 | 0.755 |
| Poorly | 270 | 93 | 177 |  |  | 242 | 119 | 123 |  |
| Location | | | | | | | | | |
| Antrum | 124 | 38 | 86 | 0.440 |  | 110 | 51 | 59 | 0.306 |
| Cardias | 150 | 54 | 96 |  |  | 137 | 68 | 69 |  |
| Corpus and fundus | 58 | 23 | 35 |  |  | 56 | 33 | 23 |  |
| Size | | | | | | | | | |
| < 5cm | 148 | 36 | 112 | 0.001* |  | 129 | 45 | 84 | 0.000* |
| ≥ 5cm | 216 | 88 | 128 |  |  | 200 | 119 | 81 |  |
| LN | | | | | | | | | |
| Yes | 103 | 46 | 57 | 0.010* |  | 88 | 55 | 33 | 0.006* |
| No | 266 | 81 | 185 |  |  | 245 | 111 | 134 |  |
| Clinical stage | | | | | | | | | |
| Ⅰ+Ⅱ | 125 | 48 | 77 | 0.271 |  | 110 | 56 | 54 | 0.787 |
| Ⅲ+Ⅳ | 248 | 81 | 167 |  |  | 227 | 112 | 115 |  |

**^a^Total case number was less than 373 owing to missing data;**

**^b^ According to the pathological classification of WHO, gastric cancer is divided into four types**

1= tubular adenocarcinoma, Papillary adenocarcinoma, and well- moderately differentiated adenocarcinoma

2= Mucinous adenocarcinoma and Mucocellular Carcinoma

3= poorly differentiated adenocarcinoma and diffuse-type cancer

4= mixed type (adenocarcinoma and Mucinous adenocarcinoma)

***Indicate statistically significant (*P*< 0.05)**

**Table S3 Univariate Cox proportional hazard model comparing expression of *miR-125b*(*AR*, *BIK*, *CASP 6*) with survival in GC**

| Variable | Overall Survival | | | |  |  | Disease-free Survival | | | |  |
| --- | --- | --- | --- | --- | --- | --- | --- | --- | --- | --- | --- |
|  | **Univariate model** | | **Multivariate model** | | |  | **Univariate model** | | | **Multivariate model** | |
|  | **HR^a^ (95%CI^b^)** | ***P*** | **HR (95%CI)** | | ***P*** |  | **HR (95%CI)** | ***P*** | | **HR (95%CI)** | ***P*** |
| Gender(Female/male) | 0.96 (0.74-1.31) | 0.804 | | |  |  | 0.95 (0.69-1.29) | | 0.731 |  |  |
| Age(≥60/<60) | 1.01 (0.77-1.32) | 0.956 | | |  |  | 1.00 (0.76-1.31) | | 0.981 |  |  |
| Smoke(Yes/No) | 1.09 (0.83-1.44) | 0.548 | |  |  |  | 1.09 (0.83-1.44) | | 0.526 |  |  |
| Alcohol drink(Yes/No) | 1.12 (0.84-1.48) | 0.455 | |  |  |  | 1.09 (0.83-1.45) | | 0.534 |  |  |
| Path | -- | 0.069 | |  |  |  | -- | | 0.050* |  |  |
| Path(2/1) | 1.45 (0.97-2.19) | 0.073 | |  |  |  | 1.54 (1.03-2.32) | | 0.037* |  |  |
| Path(3/1) | 1.60 (1.13-2.27) | 0.008* | |  |  |  | 1.63 (1.15-2.30) | | 0.006* |  |  |
| Path(4/1) | 1.39 (0.63-3.09) | 0.418 | |  |  |  | 1.34 (0.60-2.98) | | 0.47 |  |  |
| Differentiation  (poorly/well and moderately) | 1.81 (1.28-2.55) | 0.001* | |  |  |  | 1.77 (1.26-2.49) | | 0.001* |  |  |
| Location | -- | 0.033* | |  |  |  | -- | | 0.038* |  |  |
| Cardia/Antrum | 1.41 (1.02-1.95) | 0.040* | |  |  |  | 1.39 (1.00-1.92) | | 0.047* |  |  |
| Corpus and Fundus/Antrum | 0.88 (0.57-1.35) | 0.547 | |  |  |  | 0.88 (0.57-1.35) | | 0.544 |  |  |
| Size(≥5cm/<5cm) | 1.16 (0.87-1.55) | 0.303 | |  |  |  | 1.15 (0.87-1.53) | | 0.338 |  |  |
| LN(Yes/No) | 2.89 (1.98-4.22) | 0.000* | |  |  |  | 2.91 (1.99-4.25) | | 0.000* |  |  |
| Clinical stage(III+IV/I+II) | 2.72 (1.94-3.81) | 0.000* | |  |  |  | 2.75 (1.96-3.85) | | 0.000* |  |  |
| *MiR-125b* expression (high/low)^c^ | 1.48 (1.09-2.00) | 0.011* | | 1.42 (1.02-1.98) | 0.039* |  | 1.42 (1.05-1.91) | | 0.022* | 1.27 (0.98-1.66) | 0.073 |
| *Caspase-6* expression (high/low)^d^ | 0.72 (0.53-0.98) | 0.034* | | 0.72 (0.53-0.97) | 0.033* |  | 0.73 (0.54-0.99) | | 0.045* | 0.81 (0.61-1.08) | 0.148 |
| *BIK* expression (high/low)^e^ | 0.68 (0.46-1.00) | 0.050* | | 0.70 (0.49-1.00) | 0.051 |  | 0.68 (0.78-0.97) | | 0.034* | 0.73 (0.52-1.02) | 0.066 |
| *AR* expression (high/low)^f^ | 1.38 (1.03-1.85) | 0.029* | | 1.38 (1.02-1.85) | 0.035* |  | 1.42 (1.06-1.90) | | 0.019* | 1.46 (1.10-1.94) | 0.009* |

^a^HR, Hazard ratio;

^b^CI, Confidence interval

**^c^**Low expression, n=129; high expression, n=244;

**^d^**Low expression, n=186; high expression, n=139;

**^e^**Low expression, n=91; high expression, n=204;

**^f^**Low expression, n=169; high expression, n=168.

|  | Caspase-6 expression(n=325) | | | |  | BIK expression(n=295) | | | |
| --- | --- | --- | --- | --- | --- | --- | --- | --- | --- |
|  | **Total** | **Low** | **High** | ***P*** |  | **Total** | **Low** | **High** | ***P*** |
| Gender | | | | | | | | | |
| Male | 243 | 133 | 110 | 0.117 |  | 220 | 150 | 70 | 0.536 |
| Female | 82 | 53 | 29 |  |  | 75 | 54 | 21 |  |
| Age | | | | | | | | | |
| < 60 | 151 | 90 | 61 | 0.421 |  | 136 | 95 | 41 | 0.810 |
| ≥ 60 | 174 | 96 | 78 |  |  | 159 | 109 | 50 |  |
| Smoke | | | | | | | | | |
| Yes | 156 | 89 | 67 | 0.838 |  | 138 | 99 | 39 | 0.370 |
| No | 165 | 96 | 69 |  |  | 154 | 103 | 51 |  |
| Alcohol drink | | | | | | | | | |
| Yes | 186 | 108 | 78 | 0.918 |  | 166 | 112 | 51 | 0.889 |
| No | 127 | 73 | 54 |  |  | 118 | 82 | 36 |  |
| Path | | | | | | | | | |
| Path1 | 88 | 30 | 58 | 0.000* |  | 81 | 48 | 33 | 0.136 |
| Path2 | 72 | 57 | 15 |  |  | 61 | 46 | 15 |  |
| Path3 | 154 | 95 | 59 |  |  | 141 | 102 | 39 |  |
| Path4 | 11 | 4 | 7 |  |  | 12 | 8 | 4 |  |
| Differentiation | | | | | | | | | |
| Well/Moderately | 88 | 32 | 56 | 0.000* |  | 80 | 49 | 31 | 0.059 |
| Poorly | 233 | 153 | 80 |  |  | 212 | 154 | 58 |  |
| Location | | | | | | | | | |
| Antrum | 109 | 63 | 46 | 0.018* |  | 94 | 72 | 22 | 0.006* |
| Cardias | 136 | 67 | 69 |  |  | 121 | 71 | 50 |  |
| Corpus and fundus | 39 | 29 | 10 |  |  | 38 | 30 | 8 |  |
| Size | | | | | | | | | |
| < 5cm | 128 | 76 | 52 | 0.534 |  | 111 | 75 | 36 | 0.535 |
| ≥ 5cm | 188 | 105 | 83 |  |  | 176 | 125 | 51 |  |
| LN | | | | | | | | | |
| Yes | 91 | 47 | 44 | 0.172 |  | 78 | 52 | 26 | 0.487 |
| No | 230 | 138 | 92 |  |  | 213 | 151 | 62 |  |
| Clinical stage | | | | | | | | | |
| Ⅰ+Ⅱ | 110 | 63 | 47 | 0.991 |  | 93 | 63 | 30 | 0.608 |
| Ⅲ+Ⅳ | 215 | 123 | 92 |  |  | 198 | 140 | 58 |  |

**Table S6 Association of CASP6 and BIK expression with clinicopathological features of gastric cancer patients**

**Table S7 The information on the antibodies used in this study.**

| Name | Description | Product code | Company | Dilution Ratio | |
| --- | --- | --- | --- | --- | --- |
| anti-AR  anti-PARP  anti-Cleaved-PARP  anti-BAX  anti-BCL2  anti-Caspase-6  anti-DDDDK-tag  anti-BIK  Normal mouse IgG  Normal rabbit IgG  anti-Ki-67 | Rabbit, mAb^a^ | D6F11 | CST, USA | 1:1000 |  |
|  | Rabbit, mAb  Rabbit, mAb  Rabbit, mAb  Rabbit, mAb  Mouse, mAb  Rabbit, mAb  Mouse, mAb  Rabbit, PcAb^b^ | E3S4N  46D11  D64E10  Ab32503  Ab692  Ab108335  M185-3L  Sc-1070  12-349  10500C  ZM-0166 | CST, USA  CST, USA  CST, USA  Abcam, USA  Abcam, USA  Abcam, USA  MBL  Santa Cruz, USA  Millipore  Invitrogen  Zhongshan (Zsbio), Beijing, CN | 1:50  1:1000  1:1000  1:1000  1:500  1:1000  1:50  1:500  1:2000  1:2000  ready to use |  |
| Anti-β-actin | Mouse, mAb | sc-47778 | Santa Cruz Biotechnology, USA | 1:2000 |  |

^a^mAb: monoclonal

^b^PcAb: polyclonal

**Table S8**  **Primers and probes used in this experiment**

| **Primers used for *miR-125b* targets luciferase assay ( pmirGLO cloning primers)** | | | | | | | |
| --- | --- | --- | --- | --- | --- | --- | --- |
| BAK 3’-UTR | | BAK1 3’-UTR sense | | | CTAGCGGCCGCTTTGCAGTTGGACTCTCAGGGATTCTGT | | |
|  |  | BAK1 3’-UTR antisence | | | CTAGACAGAATCCCTGAGAGTCCAACTGCAAAGCGGCCGCTAGAGCT | | |
|  |  | BAK1 3’-UTR mismatch sense | | | CTAGCGGCCGCTTTGCTCAAGGACCCATGCTTATTCTGT | | |
|  |  | BAK1 3’-UTR mismatch antisence | | | CTAGACAGAATAAGCATGGGTCCTTGAGCAAAGCGGCCGCTAGAGCT | | |
| CASP6 3’-UTR | | CASP6 3’-UTR sense | | | CTAGCGGCCGCAATCCTCAGGAAATTAGATAAATT | | |
|  |  | CASP6 3’-UTR antisence | | | CTAGAATTTATCTAATTTCCTGAGGATTGCGGCCGCTAGAGCT | | |
|  |  | CASP6 3’-UTR mismatch sense | | | CTAGCGGCCGCAATCTCAGCAAAATTAGATAAATT | | |
|  |  | CASP6 3’-UTR mismatch antisence | | | CTAGAATTTATCTAATTTTGCTGAGATTGCGGCCGCTAGAGCT | | |
| BIK 3’UTR | | BIK 3’-UTR sense-1 | | | CTAGCGGCCGCGCGGCTCAGGGCGGGGCTGGCT | | |
|  |  | BIK 3’-UTR antisence-1 | | | CTAGAGCCAGCCCCGCCCTGAGCCGCGCGGCCGCTAGAGCT | | |
|  |  | BIK 3’-UTR mismatch sense-1 | | | CTAGCGGCCGCGCGGAGGCTTACGGGGCTGGCT | | |
|  |  | BIK 3’-UTR mismatch antisence-1 | | | CTAGAGCCAGCCCCGTAAGCCTCCGCGCGGCCGCTAGAGCT | | |
|  |  | BIK 3’-UTR sense-2 | | | CTAGCGGCCGCTGCTGAGGTTTTATACTCAGGTTTTTTGT | | |
|  |  | BIK 3’-UTR antisence-2 | | | CTAGACAAAAAACCTGAGTATAAAACCTCAGCAGCGGCCGCTAGAGCT | | |
|  |  | BIK 3’-UTR mismatch sense-2 | | | CTAGCGGCCGCTGCTGGTGTTTTATAAGGCACTTTTTTGT | | |
|  |  | BIK 3’-UTR mismatch antisence-2 | | | CTAGACAAAAAAGTGCCTTATAAAACACCAGCAGCGGCCGCTAGAGCT | | |
| **Primers used for *miR-125b* promoter luciferase assay (pGL3-basic cloning primers)** | | | | | | | |
| *miR-125b-1*  ARE WT | | | MIR125B1-ARE-F | | GCGGGTACCTACTAAGTGCGTTGAGGCCT | | |
|  |  |  | MIR125B1-ARE-R | | GCGACGCGTTCGGGTTTTCAAAGGAGAAGA | | |
| *miR-125b-*2  ARE WT  (ARE 2,3,4) | | | MIR125B2-ARE-F | | AGAGGTACCACATCATGGAGCAGAATATT | | |
|  |  |  | MIR125B2-ARE-R | | CACACGCGTATTTATAGACTAATTCCAGA | | |
| *miR-125b-*1  ARE DEL  (ARE1) | | | ARE1-DEL-F | | GCCTGGAATAAAAGAAGAAGAAGAAAGAAA | | |
|  |  |  | ARE1-DEL-R | | TTTCTTTCTTCTTCTTCTTTTATTCCAGGC | | |
| *miR-125b-2*  ARE DEL  (ARE 2,3,4) | | | ARE 2  deletion | ARE2-DEL-F | | | AAATGGATGATTCACCACAGGAAAACATTC |
|  |  |  |  | ARE2-DEL-R | | | GAATGTTTTCCTGTGGTGAATCATCCATTT |
|  |  |  | ARE 3  deletion | ARE3-DEL-F | | | CTAAAGTACAAATTCCGAACCACAAACAAG |
|  |  |  |  | ARE3-DEL-R | | | CTTGTTTGTGGTTCGGAATTTGTACTTTAG |
|  |  |  | ARE 4  deletion | ARE4-DEL-F | | | GAAAAGTTTTTGTTTTAACTGCAACGGTAT |
|  |  |  |  | ARE4-DEL-R | | | ATACCGTTGCAGTTAAAACAAAAACTTTTC |
| **Probes used for EMSA** | | | | | | | |
| ARE 1 | MIR125B1-F-Biotin | | | | | GAATAAAAGAAGAACAAGAAGAAGAA-Biotin | |
|  | MIR125B1-F | | | | | GAATAAAAGAAGAACAAGAAGAAGAA | |
|  | MIR125B1-R | | | | | TTCTTCTTCTTGTTCTTCTTTTATTC | |
|  | Del-MIR125B1-F-Biotin | | | | | GAATAAAAGAAGAAGAAGAA-Biotin | |
|  | Del-MIR125B1-R | | | | | TTCTTCTTCTTCTTTTATTC | |
| ARE 2 | MIR125B2-S1-F-Biotin | | | | | ATGATTCACCTGTTCTACAGGAAAAC-Biotin | |
|  | MIR125B2-S1-F | | | | | ATGATTCACCTGTTCTACAGGAAAAC | |
|  | MIR125B2-S1-R | | | | | GTTTTCCTGTAGAACAGGTGAATCAT | |
|  | Del-MIR125B2-S1-F-Biotin | | | | | ATGATTCACCACAGGAAAAC-Biotin | |
|  | Del-MIR125B2-S1-R | | | | | GTTTTCCTGTGGTGAATCAT | |
| ARE 3 | MIR125B2-S2-F-Biotin | | | | | GTACAAATTCTGTTCTCGAACCACAA-Biotin | |
|  | MIR125B2-S2-F | | | | | GTACAAATTCTGTTCTCGAACCACAA | |
|  | MIR125B2-S2-R | | | | | TTGTGGTTCGAGAACAGAATTTGTAC | |
|  | Del-MIR125B2-S2-F-Biotin | | | | | GTACAAATTCCGAACCACAA-Biotin | |
|  | Del-MIR125B2-S2-R | | | | | TTGTGGTTCGGAATTTGTAC | |
| ARE 4 | MIR125B2-S3-F-Biotin | | | | | GTTTTTGTTTTGTTCTTAACTGCAAC-Biotin | |
|  | MIR125B2-S3-F | | | | | GTTTTTGTTTTGTTCTTAACTGCAAC | |
|  | MIR125B2-S3-R | | | | | GTTGCAGTTAAGAACAAAACAAAAAC | |
|  | Del-MIR125B2-S3-F-Biotin | | | | | GTTTTTGTTTTAACTGCAAC-Biotin | |
|  | Del-MIR125B2-S3-R | | | | | GTTGCAGTTAAAACAAAAAC | |
| **Primers used for ChIP-qPCR** | | | | | | | |
| ARE 1 | ChIP-MIR125B1-F | | | | TAGCCAGCCATCTACCTTGC | | |
|  | ChIP-MIR125B1-R | | | | AATTCCCCTCCATCTTCGCA | | |
| ARE 2 | ChIP-MIR125B2-S1-F | | | | GGGTGTGGTACTAGGGTGGA | | |
|  | ChIP-MIR125B2-S1-R | | | | AGTGGAATCTGGCTCTTCTGA | | |
| ARE 3 | ChIP-MIR125B2-S2-F | | | | TGTGGCCAGGACTTTTACAT | | |
|  | ChIP-MIR125B2-S2-R | | | | AATACACAACCCCAATCCTGT | | |
| ARE 4 | ChIP-MIR125B2-S3-F | | | | TGCTGTCTGAATAGTTTGGAGA | | |
|  | ChIP-MIR125B2-S3-R | | | | TTGCTTCCCATTACACTGCT | | |

The captions for Table S2, S4, S5 provided as a separated Excel file

**Table S2:**  The top 100 genes list with high attribution score to gender difference identified from the five GC cohorts

**Table S4:**  The list of the predicted 178 targets of *miR-125b* in GC samples

**Table S5:**  The pathway enrichment analysis results of the predicted targets of *miR-125b* in GC

The captions for Data S1

**Data S1**: STR profiling reports of GC cell lines (HGC-27, MGC-803, SGC-7901)
